# A Little Bird Told Me… Nutri-Score Panoramas from a Flight over Europe, Connecting Science and Society

**DOI:** 10.3390/nu15153367

**Published:** 2023-07-28

**Authors:** Alice Stiletto, Leonardo Cei, Samuele Trestini

**Affiliations:** Department of Land, Environment, Agriculture and Forestry, University of Padova, 35020 Legnaro, Italy; alice.stiletto@unipd.it (A.S.); leonardo.cei@unipd.it (L.C.)

**Keywords:** front of pack, nutritional label, topic model analysis, consumers preferences, systematic literature review, Twitter analysis

## Abstract

Within the Farm to Fork Strategy, the European Commission ask for a unified Front Of Pack nutritional label for food to be used at the European level. The scientific debate identified the Nutri-Score (NS) as the most promising candidate, but within the political discussion, some Member States brought to attention several issues related to its introduction. This misalignment led to a postponement of the final decision. With the aim to shed some light on the current stances and contribute to the forthcoming debate, the objective of the present work is to understand to what extent scientific research addresses the issues raised by the general public. We applied a structural topic model to tweets from four European countries (France, Germany, Italy, Spain) and to abstracts of scientific papers, all dealing with the NS topic. Different aspects of the NS debate are discussed in different countries, but scientific research, while addressing some of them (e.g., the comparison between NS and other labels), disregards others (e.g., relations between NS and traditional products). It is advisable, therefore, to widen the scope of NS research to properly address the concerns of European society and to provide policymakers with robust evidence to support their decisions.

## 1. Introduction

Currently, overnutrition is the main nutritional issue at the global level, as 24.1% of adults are overweight and obese—and only 5.8% are underweight [1]. To reduce and prevent this issue, Front-Of-Pack labels (FOPLs) have been widely used both at the global and European levels to improve the nutritional and health habits of the population [2]. These labels, providing concise and easy-to-understand information about the nutritional profile of foods on the front of the pack, have a double goal: to help consumers to identify the overall nutritional quality of food, thus guiding them towards healthier food choices [3] and to encourage food industries to reformulate and improve their products [4].

**Figure 1:**
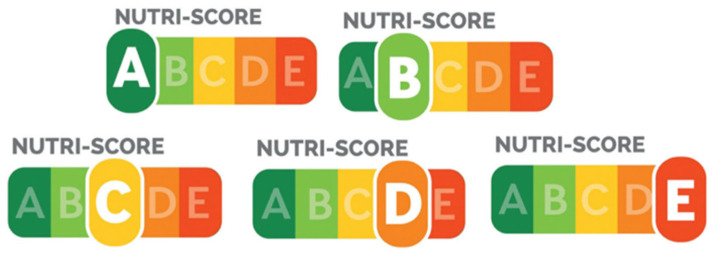
Nutri-Score labels.

At the European level, multiple FOPLs currently co-exist, such as nutrient-specific labels (e.g., Reference Intake), endorsement schemes (e.g., GreenKeyhole), and summary labels (e.g., Nutri-Score), which are adopted on a voluntary basis by EU countries and firms. However, as FOPLs are not mandatory yet, food industries can take advantage of its adoption, using the labels only on products whose sales value could be increased by use of the FOPLs [5]. To overcome this issue, the Farm to Fork (F2F) strategy stresses the need to make the use of FOP nutritional labelling mandatory on pre-packed foods, using a harmonized standard across the EU. The Nutri-Score (NS) is the most promising FOP candidate to be used, being considered the most efficient in helping consumers to discriminate products according to their nutritional profile [6,7,8]. The NS is a five-step colour-graded nutrition label (Figure 1), ranging from the healthiest category, the dark green (category A), to the unhealthiest one, the red one (category E). As a summary label, it provides an overall assessment of a food’s nutritional value, considering favourable (i.e., content of fruits and vegetables, fibre, protein, nuts, rapeseed, and olive oil) and unfavourable nutrients (i.e., content of calories, fat, sugars, and salt) for classifying foods into one out of the five categories.

Despite being currently adopted in several European countries, the NS is stimulating an active debate, while it has faced (and is still facing) oppositions. In France, after its first proposal in 2013 (which led to the final adoption in 2017), an outcry was raised, especially from agro-food companies [9]. The subsequent request of the EU (within the F2F strategy) to use it on a mandatory basis in all EU countries widened the debate to other Member States. In Italy, the NS adoption is a recurrent theme of the agricultural political debate, where the national government supports the major agro-food firms [10] in their claim of the NS as a penalizing tool for Mediterranean and traditional products [10,11], including wines (available at: https://foodmatterslive.com/article/nutri-score-proposal-alcohol-lowest-ranking-grade-criticised-france-italy/; accessed on 14 June 2023). Similarly, in Spain, where the NS was adopted in 2021, concerns were repeatedly raised about supposed inconsistencies in the classification of some traditional products, such as olive oil [12] (the NS algorithm was modified at a later stage to positively value the nutritional qualities of olive oil).

In light of these discussions, there is a clear need, at the EU level, to shed some light on the contrasting positions existing within the European context, to reach a general agreement among Member States. This is all the more important considering that the European Commission has recently postponed the presentation of the proposal of a single FOPL to 2024 (i.e., to the next European legislature) because of contrasts between EU countries and the lack of sufficient data to support the label. In addition, to date, the 150 papers that focus on the NS label are not equally distributed across Europe. Specifically, France (20.5% of publications), which is the country where the NS was initially adopted, has produced two times the publications of other countries, such as Spain (10.9%) or Italy (7.0%). Considering this, the scientific literature could be in some way biased, focusing only on the aspects related to the NS that are more interesting for the countries in which the NS topic is more addressed. However, to decide what FOPL to adopt at the EU level, the European Commission needs to have a complete overview of the NS topic, evaluating all its aspects. In this respect, it is important to take an informed policy decision, to gain insights about the most relevant aspects raised by citizens and researchers. In line with this consideration, in this study we aim to provide an overview of the Nutri-Score discussion in Europe, highlighting to what extent scientific research has addressed the concerns raised by public opinion. To do so, we aim to answer the following research questions (RQ):

RQ1: What are the topics raised by the public debate on the NS label in different EU countries?

RQ2: To what degree does the scientific research on NS address all the aspects that have emerged from the public debate?

The data collection process and the methodological approach used to analyze textual data from the two sources (i.e., Twitter and the scientific literature) are detailed in the next section. In Section 3, we report the results separately for Twitter and the literature analysis and, within the former, for each considered country. A thorough and wide-ranging discussion is provided in Section 4, where comparisons of country-specific NS discourses are critically illustrated, while public and scientific debates are confronted. Some conclusions are provided at the end of the manuscript, stressing the implications of our results for both policy action and scientific research.

## 2. Materials and Methods

To answer RQ1, a topic-modeling analysis has been conducted on tweets posted on Twitter (RQ1) in four different EU countries (France, Germany, Italy, and Spain). Indeed, as Twitter is the social network platform most used by institutions, industries, and organizations to share information or to discuss legislations [13], it is the most suitable tool to catch the public discussions on NS. Several scholars have already analyzed tweets’ content for comparing experts’ opinions on specific topics, such as cardiovascular diseases [14], or to understand public opinion on hot topics, such as COVID-19 in 2020 [15]. In addition, Ola and Sedig [16] and Pershad et al. [17] used Twitter analysis in health-related contexts, and Septia Irawan et al. [18] used it within the policy framework to understand the perceptions and sentiment of public discourse on FOPLs in the EU.

On the other hand, to understand if the scientific literature has covered all the aspects that have emerged from the public debate, thus providing the European Commission with an appropriate overview on the NS topic, a comparison between the topics that have emerged from the tweets analysis and the scientific research has been conducted (RQ2). To reach this objective, a systematic literature review of papers dealing with the NS issues and a topic-modelling analysis on them have been performed.

To properly compare the scientific literature with the Twitter debate on NS, it is necessary to adopt consistent and homogeneous methodological strategies both to retrieve the initial material (i.e., scientific documents and tweets) and to analyze its content. In the following subsections, we first describe the process of data collection and the pre-processing of the textual material, and then we provide a brief overview of the topic-modeling technique used to identify the main topics. All statistical analyses were performed using the R software (version 4.2.2).

### 2.1. Data Collection and Pre-Processing

#### 2.1.1. Tweets

To assure consistency with the literature analysis (Section 2.1.2), the analysis of the Twitter data was conducted on tweets mentioning the words “Nutriscore” or “Nutri-score” that were posted between January 2017 and January 2023. Before 2017, tweets about the NS were in fact scanty. Retweets are excluded from the analysis, a procedure also adopted in other studies analyzing the contents of tweets (see, for example, [15,19]). Specifically, while retweets might signal agreement with (or sharing of) someone else’s opinion, tweets of popular users (e.g., politicians, influencers, celebrities) are more likely to be retweeted than tweets from ordinary users. As such, the inclusion of retweets in our analysis might have led to an overrepresentation of the interests of relatively few individuals, with the subsequent introduction of a bias in the results.

In order to work with a sufficiently high number of tweets and thus conduct a meaningful statistical analysis, we decided to restrict the scope to the four countries with the highest number of tweets about the NS: France, Germany, Italy, and Spain. In this respect, the country of origin of the tweets was determined on the basis of the tweet language.

The assignment of the location of tweets based on the language in which they were written is a delicate step and it therefore deserves further attention. Twitter can provide geolocation information for tweets, but only few users activate this specific function. As a result, the majority of tweets cannot be linked to a specific country of origin, hence the decision to rely on the tweets’ language.

It is important to note that the use of the tweet language is not free of possible biases. Specifically, two kinds of errors are possible:

(i) False positives: a tweet is attributed to a certain nationality (because it is written in the native language of that country) when it is in fact coming from another country;
(ii) False negatives: a tweet is not attributed to the correct nationality when it is in fact coming from that country, because it is not written in the native language of that country.

Both types of errors are more frequent for languages that are widely used outside their countries, with English representing the major concern.

False positives can also appear, however, for the languages considered in our analysis: French is used in Belgium, Switzerland, Canada, and some African countries; German in Austria and Switzerland; Italian in Switzerland; and Spanish in Latin America. In the case of languages used in neighboring European countries, the main country (France, Germany, Italy) always has a far larger population, assuring the attribution errors are minimal. For languages used outside Europe, on the other hand, the assurance is given by the topic addressed. NS is in fact, to date, a subject debated almost exclusively in Europe, where it was devised and implemented. The number of tweets from major non-European countries was assessed using the Twitter geolocation function and compared with geolocated tweets from the four countries included in the analysis. Overall, the United States, Canada, Brazil, Argentina, China, Japan, India, and Australia accounted for 40 tweets, while 1497 tweets were posted in the four European countries.

Conversely, to assess the relevance of false negatives, we retrieved the geolocated tweets from the four countries and we counted the number of tweets written in the non-native language. As reported in Table 1, in three of the four countries, tweets posted in the native language accounted for more than 80% of the tweets, while a lower share was observed in Italy. It is important to note that false negatives, while they might still introduce some bias reducing a country’s population of tweets, do not cause a misallocation of tweets.

The use of the language criterium to assign nationality to tweets provided 71,089 original tweets. These tweets were pre-processed following a procedure drawn from Lyu et al. [19]. Specifically, we removed URLs, non-ASCII characters and numbers, and we dropped similar tweets. Indeed, similar and duplicate tweets stem, in most of the cases, from retweets posted without the specific retweeting function, which therefore do not allow them to be identified as retweets in the first place. The similarity between tweets was assessed by computing the cosine similarity for each pair of tweets based on the document-term matrix, a matrix where rows represent tweets, columns correspond to terms, and single cells contain 1 if a term is present in a tweet and 0 if it is not. The cosine similarity is given by the dot products between two rows. When the similarity between two tweets was higher than 90%, only one of them was retained. This process led to four national databases consisting, overall, of 65,723 tweets.

#### 2.1.2. Scientific Literature

The collection of scientific documents was performed following the PRISMA (Preferred Reporting Items for Systematic reviews and Meta-Analysis for protocols) guidelines [20]. The first step of the protocol consists in planning the review, whose pivotal point is the definition of the objective. In this respect, as discussed in the introduction, our aim is to have a broad view of the scientific literature investigating the NS label, irrespective of the specific scientific subject area.

In line with this objective, we decided to begin the second step (i.e., conducting the review) choosing a loose search string:

TITLE-ABS-KEY (Nutriscore OR Nutri-score)

The search was performed in the two largest scientific databases, Scopus and Web of Science, in January 2023, considering only published original articles written in English (notes, letters, conference papers, editorials, and reviews were excluded). Although scholars usually extend the research to other sources of data, not necessarily scientific (see for instance [21]), Scopus and Web of Science are considered the most comprehensive databases of high-quality peer-review articles [22,23,24]. This initial step provided 329 articles. This set of articles was reduced, through successive phases, to 150 articles. Specifically, 156 duplicate articles deriving from the merging of the two sources (Scopus and Web of Science) were initially discarded. After reading the titles and the abstracts of the remaining 173 articles, 23 additional documents were excluded. Of the 23 excluded papers, 2 are additional reviews not excluded from the initial search, 17 are medical articles referring to a homonymous nutritional screening tool for oncological patients [25], and 4 simply do not deal with the NS.

### 2.2. Data Analysis—Topic Modeling

The analysis of the contents of tweets and of the scientific literature was performed in R using structural topic modeling (STM) (stm package, [26]). STM is a quantitative text analysis technique that allows for the retrieval of underlying topics from a corpus of documents and that is increasingly exploited in several research fields (some examples are [27,28,29]). Specifically, the STM was applied to five corpora separately: the corpus of the abstracts of scientific articles and the four national corpora of tweets. The STM models were estimated on tweets in their original language. English translation was used at a later stage only to interpret the results.

The main advantage of STM and similar text analysis techniques consists in the ability to deal with a large number of documents that might be hardly tractable by one or a few researchers. In our case, this is particularly valuable for the analysis of tweets, while the size of the scientific literature corpus would have allowed the performance of a standard literature review. However, a robust comparison between different text corpora requires the analysis of them with identical methodologies. In addition, using such a technique proves even more useful when the objective is to compare different sets of documents, since it assures the removal of any possible bias that might be inadvertently introduced by the discretion of the researcher. Compared with other quantitative text analysis techniques, STM allows a document to include multiple topics, thus better resembling the complexity of scientific communication and public opinion.

STM was devised by Roberts et al. [30,31] and is part of a family of techniques whose objective is to extract from a corpus of documents its content. This content is represented by the topics, which are identified as latent structures in the corpus. The STM relies on the assumption of a specific generative process for the corpus at hand.

The generative process explains how the corpus came to be created, starting from the selection of each single word of each document. For clarity, we provide a brief summary of the process. First, the total number of words contained in a document *d* (*N_d_*) is extracted from a Poisson distribution. Then, given *K* topics, for each document of the corpus a vector of topic proportions (*θ_d_*) is extracted from a logistic normal distribution. This vector represents the proportion of a document that addresses each *k* topic, which is commonly defined as the topical prevalence. As a third step, based on *θ_d_*, the topic of each *n*th word is determined. The last step consists of the drawing of each specific *n*th word. Each topic is characterized by a specific word distribution, which is called the topical content. The *n*th word is thus drawn from the distribution of the relative topic [31].

Exploiting a Bayesian approach, the STM walks this generative process backwards and, starting from the words observed in the documents, retrieves the topical content and the topical prevalence of each topic.

The characteristics of the assumed generative process confer on the STM some interesting properties: (i) each document is considered a mixture of topics; (ii) correlation between topics can be estimated; (iii) covariates can be used to model topical prevalence and/or topical content. The last aspect is particularly innovative, since it allows either the proportions of the topic in the corpus (topical prevalence) or the words used to identify a topic (topical content) to vary according to documents’ pre-specified characteristics.

With respect to our analysis, the first step was to structure the model, which included the selection of the covariates. For the four models set for the analysis of tweets, we included time as a covariate for modeling topical prevalence, using splines to account for possible non-linear relationships. Time is defined as the month when a tweet was posted. We decided not to include time as a covariate in the literature model. Despite the fact that the topics addressed by the scientific literature might vary over time, considering the time needed to prepare a scientific paper and to go through the whole publication process, we deem the time span of the analysis (2017–2023) too short to highlight any meaningful trend in the published articles.

The second step entails the decision of the number of topics for each model. In fact, while STM infers autonomously the content of the topics, their number must be specified in advance by the researcher. The selection of the optimal number of topics was performed estimating several models with different numbers of topics and then analyzing the average exclusivity (i.e., the specificity of each word to a given topic) and semantic coherence (i.e., probability of a set of words to occur together in the same document) measures of each model [26,32]. The best model is the one that scores high in both metrics, but where neither of the two dominates the other [26]. When this criterium alone was not sufficient to uniquely identify an optimal model, we restricted the analysis to the best-performing models, computed the overall average values of exclusivity and semantic coherence across the models, and selected the model with the highest share of topics with a value of both metrics above the respective average.

The last intervention of the researcher is the naming of the topics. Since STM returns the topics as words distributions, the researcher needs to infer the content of the topic and assign it a name. This is usually achieved by either analyzing the word distributions or the most representative documents of a topic. Adopting this second strategy, we selected, for each topic in each model, the documents in which that topic had a prevalence higher than 75% and, based on their content, we named the topic. To improve the consistency in the identification of the name and the content of a topic, we followed the procedure in Lyu et al. [19]. Two authors independently analyzed half of the representative documents and determined the name of the topic through group discussion. Afterwards, the third author checked the consistency of the name with the content of the most representative documents and the final name for the topic was finally selected, after additional discussion when needed.

## 3. Results

### 3.1. Twitter Analysis

As reported in Table 2, the search identified 65,723 tweets discussing NS in the four countries considered in the analysis. Weighting the number of tweets by the number of Twitter users shows that the NS topic is more popular in France, while it is relatively less debated in Germany. The estimates in Table 2 should be considered as indicative, as figures on the Twitter penetration in each country appear to be uncertain. The number of Twitter users were retrieved from web searches (available at: https://www.statista.com/statistics/242606/number-of-active-twitter-users-in-selected-countries/; https://business.trustedshops.it/blog/gruppi-utenti-social-media#:~:text=Con%204%2C79%20milioni%20di,uomini%20e%20il%2030%25%20donne, accessed on 17 June 2023) and refer to 2022.

**Figure 2:**
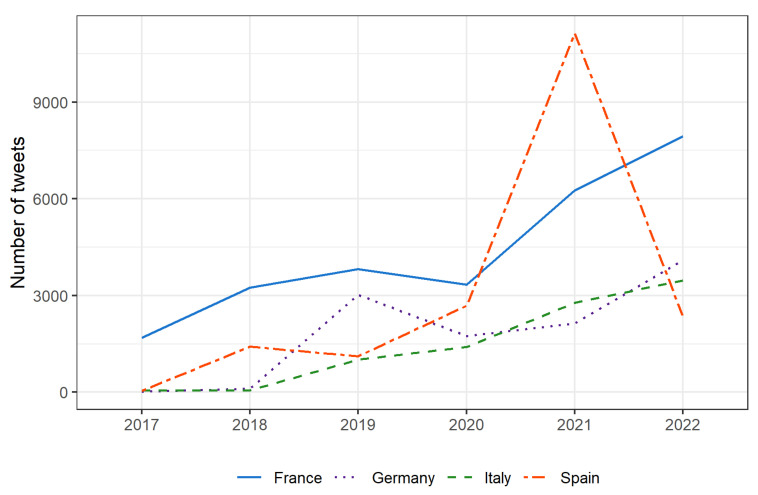
Yearly number of tweets by country.

Figure 2 presents the yearly number of tweets in each country. In this respect, different temporal patterns can be observed in the four countries, despite an increasing trend being observed everywhere. In France, the ‘homeland’ of NS, the interest of Twitter users for the topic was relatively high and constant from 2018 to 2020, despite a sharp increase being observed in the last two years with a peak in 2022. Germany and Spain are characterized by some peaks (in 2019 and in 2022 in Germany and in 2021 in Spain), while Italy displays a more constant growth.

#### 3.1.1. Italy

**Figure 3:**
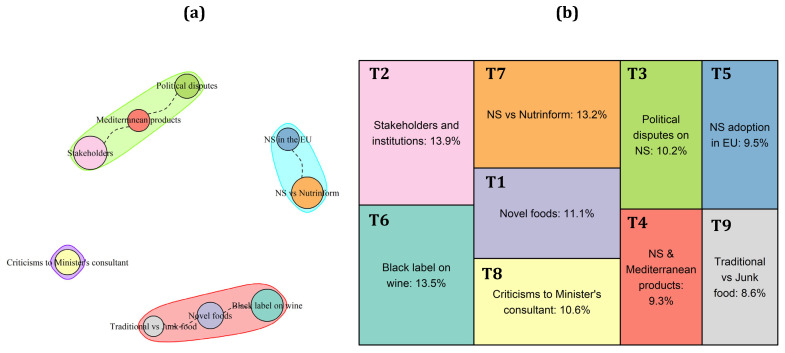
Topics’ correlation patterns (**a**) and prevalence (**b**) of the topics in the Italian corpus of tweets.

As described in Table A1 and represented in Figure 3b, nine topics emerged from the tweets analysis in Italy. Most of them describe the Italian’s contrasting position on the NS adoption (T5: “NS adoption in EU Countries”) from both a scientific (T7: “NS calculation system and comparison between NS and Nutrinform”) and a political point of view (T2: “Role and position of Stakeholders and Institutions towards NS”; T3: “Political disputes on NS”; T8: “Criticism to the Health Minister’s consultant—Walter Ricciardi—for supporting the NS system”). Specifically, different topics deal with a possible negative effect of the NS adoption on Mediterranean products, considering foods (T1: “Debate on novel foods and NS”; T4: “Implications of NS adoption for the Mediterranean products”; T9: “Criticism for NS values given to Traditional vs. Junk/Processed foods”) and wine (T6: “Position against the black label on wine).

The NS adoption (T5) in Italy seems to be a strongly debated topic, especially in recent years. Looking at the contents of the tweets, general opposition to the NS emerges, so much so that 13.2% of the corpus is dedicated to the comparison between NS and Nutri-Inform battery, the FOPL proposed by the Italian Ministry of Agriculture to the European Commission and officially presented in February 2022 as an alternative to the NS. However, the main concern of Twitter users in this country seems to be related to a possible negative effect of the NS adoption on typical products of the Mediterranean diet (T4) and on traditional products (T9). These considerations stem from the evidence that most of the high-value PDO and Protected Geographical Indication (PGI) products, such as Parmigiano Reggiano PDO, Mozzarella di Bufala Campana PDO, or Prosciutto di Parma PDO, are assigned a negative grade by the NS system (available at: https://www.ansa.it/canale_terraegusto/notizie/prodotti_tipici/2022/03/15/nutriscore-a-rischio-10-piatti-simbolo-con-i-formaggi-dop_965ef50b-0280-48a5-97f5-317c6782401b.html#:~:text=In%20pratica%20tutti%20i%20formaggi,Parmigiano%20Reggiano%20e%20Pecorino%20Romano, accessed on 22 June 2023), as is also widely acknowledged in T4. This negative sentiment is strengthened by the fact that some ultra-processed foods, generally considered as low-quality products, received positive NS values (T9). The same goes for Novel foods, such as insect-based products (T1), which are considered low-quality products by Italian users and not in line with the national culinary traditions. Following the same path, 13.5% of the corpus contains opinions of consumers and politicians towards the possibility to label wines and other alcoholic beverages with a “black F score”, in order to stress the negative effect of alcohol consumption on health, independently of the dose (T6).

Compared with other countries, tweets in Italy are strongly linked to political debates (T2; T3; T8), reflecting the strong position of the Italian government (T3), politicians, and stakeholders (T2) against the NS adoption. Tweets in T8 stress these aspects, showing how the favorable position of the Health Minister’s consultant for the NS adoption has caused such a stir among politicians and citizens.

Looking at the topic’s correlation patterns (Figure 3a), three different clusters emerged. The green one clearly represents the sentiment of national identity that drives the NS discussion in Italy, describing the possible negative effect on the Mediterranean diet products (T4), along with politicians’ (T3) and stakeholders’ (T2) positions towards this system. The red cluster collects all the tweets dealing with Italians’ concerns about the NS algorithm, considering the contrasting evaluation given by this system to novel (T1) and ultra-processed foods (T9) with respect to traditional ones, including geographical indications (T9) and wines (T6). The light blue cluster represents instead the “objective side” of the discussion, which include both considerations about the spreading of NS throughout Europe (T5) and comments about the Italian alternative to the NS label (T7). Finally, tweets discussing the very specific topic T8 stand alone.

#### 3.1.2. France

**Figure 4:**
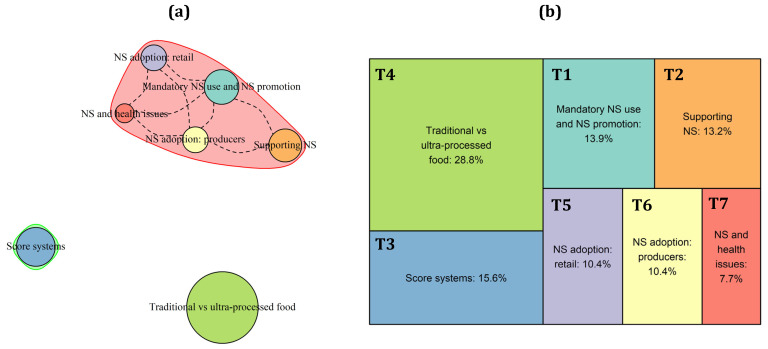
Topics’ correlation patterns (**a**) and prevalence (**b**) of the topics in the French corpus of tweets.

The analysis of tweets in France yielded seven topics, as described in Table A1 and reported in Figure 4b. Three of them (T1: “Health improvements through mandatory use and promotion of the NS”; T6: “Using the NS to improve transparency: pressures on producers”; and T7: “NS for contrasting health-related issues”) deal with positive aspects of the NS labelling, one is focused on describing some inconsistencies in the algorithm (T4: “NS vs. traditional and industrial or ultra-processed foods”), two describe the adoption of the NS (T5: “NS adoption in retail chains”) and the contrasting positions of industries (T2: “Supporting NS: lobbies hinder the adoption of NS”), and the last one deals with new score systems inspired by the NS (T3: “New score systems inspired by the NS).

In broad terms, results underlined that, according to the French twitter users, the NS adoption allows consumers to be more aware about the nutritional content of foods (T1), pushing them towards healthier food choices and thus reducing risks of health-related issues, such as cancer (T7). Indeed, the adoption of NS was strongly desired by French consumers, such that even the most reluctant producers and food industries bowed to the common will (T2; T5). However, the major share of tweets (28.8%) regards some critical issues related to NS (T4). According to these Twitter users, the algorithm underlying this labelling poorly classified some products, such as the Protected Designation of Origin (PDO) and generic cheeses or beef, while promoting some ultra-processed foods, generally perceived as unhealthy due to the high product processing. Nevertheless, the system seems to be particularly appreciated in France, so much so that new labels that are similar to the NS have been proposed in recent years to measure, for instance, cybersecurity or corporate social responsibility.

Looking at the topic correlation patterns (Figure 4a), we can appreciate that most of the topics are highly correlated to each other (red squared), underlining some overlapping discussions among them. Indeed, all these topics deal with positive aspects related to the NS and its adoption. On the contrary, tweets regarding the debate on the negative NS evaluation given to traditional or ultra-processed foods (green dot) or those focusing on other score systems that are similar to NS (blue dot) seem to stand alone.

#### 3.1.3. Germany

**Figure 5:**
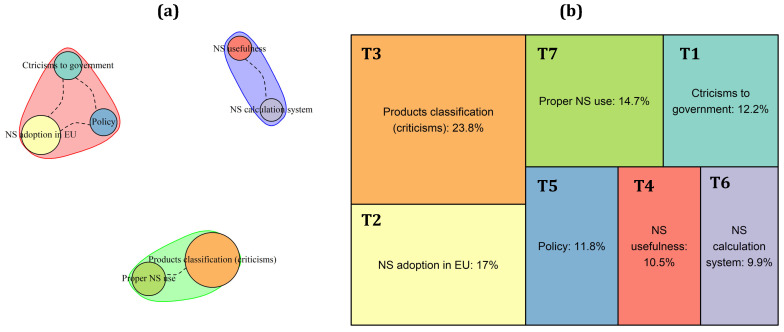
Topics’ correlation patterns (**a**) and prevalence (**b**) of the topics in the German corpus of tweets.

From the tweets’ analysis in Germany, seven topics emerged, as described in Table A1 and represented in Figure 5b. Some of them deal with technical (T7: “How to properly use the NS”; T6: “Insights on the NS calculation system”) and political (T5: “NS in the policy agenda”; T1: “Criticisms to the German Minister of Food and Agriculture—Julia Klöckner—for opportunistically not supporting the NS”) aspects linked to NS adoption (T2: “NS adoption in EU”), while others clearly adopt a judgmental perspective, stressing either the positive (T4: “Usefulness and positive aspects of NS”) or negative (T3: “Criticisms towards NS classification of products”) aspects of the NS.

In 2020, Germany adopted the NS label on a voluntary basis (T5), following the forerunner countries, such as France and Belgium (T2). This adoption has been positively welcomed by German consumers, as the NS is considered a simple and easy-to-understand label (T4), in such a way that in 12.2% of the corpus of tweets the then-Minister of Food, Julia Klöckner, is accused of having somehow hindered the adoption of this system, hiding a study reporting its benefits (available at: https://www.bmel.de/SharedDocs/Downloads/DE/_Ernaehrung/Lebensmittel-Kennzeichnung/MRI-finaler-Bericht-Naehrwertkennzeichnung.pdf?__blob=publicationFile&v=2, accessed on 17 June 2023). However, as previously discussed for the Italian and French cases, a good chunk of Twitter users (23.8%) question the calculation system behind the NS (T3), as it penalizes some product categories while promoting others, without distinguishing between different products within the same category. Some users argue that the NS does not consider some elements important for the human organism, such as vitamins, even if it appears useful for providing a general idea of the overall nutritional quality of a given product (T7). Even more than in other countries, German Twitter users seem to have contrasting positions towards the NS, with some of them strongly supporting the label and others standing against this oversimplified system (T6).

This is reflected in the topics’ correlation patterns (Figure 5a), which return three different clusters. Two of them can be distinguished on the basis of the general sentiment they convey. In the red cluster (T1, T2, T5), whose users might be identified as “NS lovers”, NS is viewed in a quite positive light. Conversely, in the green cluster (T3, T7), whose users can be named “NS faultfinders”, attention is brought to possible flaws in the NS system, whilst also discussing how to properly use and interpret this tool. Finally, the blue cluster (T4, T6), from a sentiment perspective, is more neutral in nature, its scope being limited to the provision of information about how the NS system works and how this determines its usefulness.

#### 3.1.4. Spain

**Figure 6:**
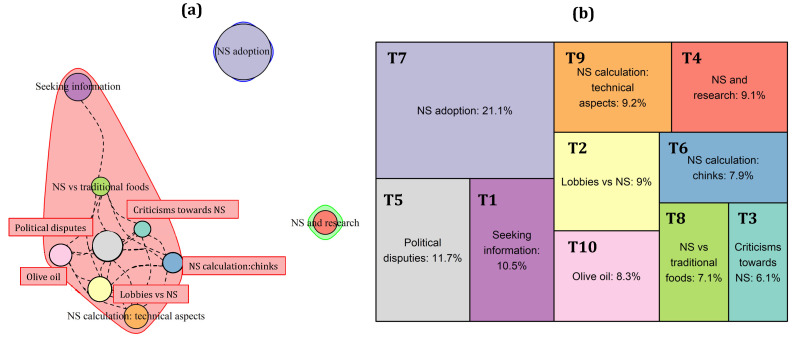
Topics’ correlation patterns (**a**) and prevalence (**b**) of the topics in the Spanish corpus of tweets.

In Spain, the NS adoption has been greatly discussed, with ten topics emerging from the Twitter analysis (Figure 6b). Indeed, the NS adoption in Spain (T7: “NS adoption”) has been widely debated, adopting either political (T5: “Political slip-ups on the NS adoption”), supply (T2: “Multinational companies against the NS adoption”), and demand perspectives (T1: “On the NS debate: seeking information”); whether scientists seem to support this label (T4: “Research support the NS”); or different criticisms of the calculation system (T3: “Criticisms towards the NS system”; T6: “NS calculation: possible chinks in the system”; T9: “NS calculation: technical aspects”), especially for undervaluing traditional Spanish products, such as the Hibernian ham (T8: “NS vs. traditional foods”) or olive oil (T10: “NS vs. olive oil -and other traditional products-”).

Spain was one of the first supporters of the NS label within the European context. Despite the Spanish government’s intention to implement it since 2018, the official adoption of the label took place only three years later, in 2021, when more than 60 Spanish scientists and nutrition professionals published a manifesto (available at: https://www.agropopular.com/manifiesto-contra-nutriscore-180221/, accessed on 23 June 2023) in support of the implementation of the NS (T4), which is considered an effective tool to guide consumers towards healthier food choices (T7). Producers had suffered pressure from consumers, who asked major food companies to adopt this labelling system, in aid of greater transparency (T2). However, as seen for the other countries, inconsistencies in the calculation system are also brought to the fore in Spain (T6), especially for not considering the meal as a whole—and rather evaluating the single ingredients—or for classifying some ultra-processed foods as the healthiest option (T6; T9). Along with this aspect, 6.1% of the corpus of tweets describes the general discontent of some Twitter users (T3) with respect to this label, which is considered too simple and not able to catch the real nutritional value of the products (T9). This is particularly true if traditional foods are considered (T8; T10), as they are highly penalized by the NS algorithm, with some industries proposing to exclude olive oil from the NS labelling (T10). In light of these controversies, some Twitter users suggested conferences and/or podcasts to follow in order to understand more in-depth what is behind the NS (T1) system, especially after the change in course of the Spanish government (available at: https://www.cope.es/actualidad/noticias/nutriscore-gana-espacio-super-mientras-gobierno-debate-regula-20211114_1616973, accessed on 23 June 2023), which, in 2021, lashed out against the French system after noting that extra-virgin olive oil (of which Spain is the world’s leading producer) is classified as a non-healthy product (T5).

Figure 6a clearly highlights the interlinkages between most of the topics. Indeed, in the Spanish case, there are no well-defined clusters of topics, as found for Italy or Germany, and to some extent in France. After the NS adoption, following the scientific evidence on the subject, several talking points seemed to be put on the table, all somehow interrelated.

### 3.2. Literature Analysis

The scientific literature on NS is, as is the topic it addresses, relatively new. The first two papers appeared in 2017, but in six years the strand grew to reach the one hundred and fifty articles included in our analysis. This trend is similar to what was observed in the tweets, and a similarity between the two debates was also observed when considering the geographical aspect. According to Scopus’s statistics, most of the scientific articles on NS are in fact produced in France (20.5%), followed mainly by other European countries.

**Figure 7:**
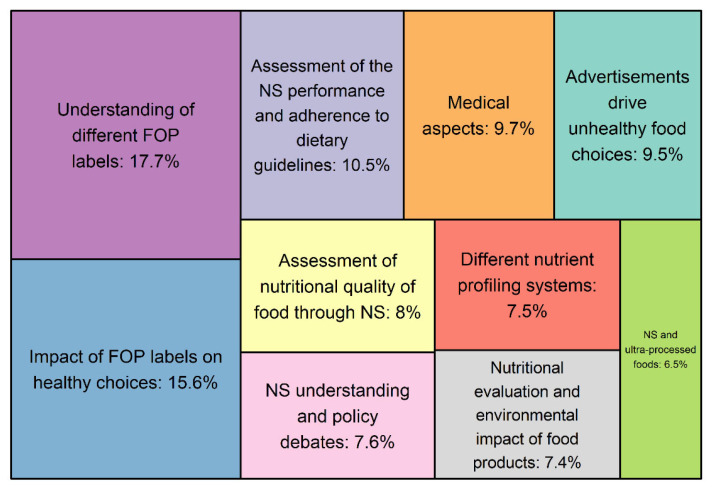
Estimated topic prevalence in the corpus of scientific abstracts.

The best STM model to describe the literature corpus is the one with ten topics, which are reported in Figure 7. In Table A2 in Appendix B, we also report, for each topic, the ten most representative terms and three titles that are among the most exemplary documents for the topic (i.e., documents where the prevalence of the topic is highest), and the references of the documents where the topic constitutes at least 25% of the abstract. In contrast to what was observed for the Twitter analysis, no interesting correlation was observed between the topics. In this respect, a role is likely played by the low number of documents in the literature corpus.

According to the model results, the most prevalent topic in the NS literature was “Understanding of different FOP labels”, which constitutes 17.7% of the corpus. The most exemplary documents of this topic usually compare different FOPLs in terms of understanding and preference by consumers. Overall, most of them agree in identifying the NS as the most understandable FOPL and the one that helps consumers the most in making healthier food choices [6,33,34]. However, some works detected that this advantage of the NS is not linked with a higher appreciation of this label compared with others [35,36,37,38]. For example, in comparing the NS with the Nutrinform label, Mazzù et al. [39] observed that Italian consumers consider the former too uninformative.

Other topics are related to the role of NS in the market and its relationship with consumers. Among these, “NS understanding and policy debates” (7.6% of the corpus) is similar to the previous one, despite focusing almost exclusively on the NS (instead of comparing multiple FOPLs). Some papers within this topic also assessed the knowledge and support for the NS among consumers and stakeholders, with mixed results according to the country where the study was based. For example, in Italy, the awareness of the NS among medical professionals is low [40], while other stakeholders are against its adoption [10]. Conversely, in France, a good amount of support is present for this label [10], while its knowledge increased over time [41].

The assessment of the knowledge and understanding of NS and FOPLs is brought to a further level in “Impact of FOP labels on healthy choices” (15.6%) and “Nutritional evaluation and environmental impact of food products” (7.4%). In both topics, in fact, the focus shifts to the impacts of NS and similar labels on food choices, thus investigating how these labels can actually modify the purchase behaviour of consumers. The former topic is characterized by the specific evaluation of the NS label while, when multiple labels are considered, this is performed in a more comparative flavour. Most of the studies associated with this topic found positive effects of the NS on the healthiness of actual purchases [42,43,44]. A recurrent finding, however, is that NS succeeds in increasing the purchase of healthy products, but it does not alter the purchase of unhealthy ones [45,46,47,48]. Studies related to the latter topic, on the other hand, tend to assess the effect of multiple labels when added together in the same product. The NS seems not to lose its effectiveness in promoting healthier food choices when other quality labels are displayed on the product [11,49,50].

While the NS is meant to drive healthier food choices, the ultimate goal is to improve, through these choices, the health of individuals. In this respect, studies focusing on “Medical aspects” (9.7%) assess whether healthy diets (where healthiness is defined according to the NS) have positive impacts on several health aspects and diseases, finding associated reductions in long-term mortality [51], kidney function decline [52], or obesity [53], among others.

A couple of the identified topics have a more technical flavour, focusing mainly on the algorithm used to obtain the NS. One of them, “Assessment of NS performance and adherence with dietary guidelines” is related to studies that verify how the NS classification performs when contrasted with specific diets. In this respect, the NS has been found to be in line with the Mediterranean diet [54], and with the Dutch, German, and Slovenian dietary guidelines [55,56,57]. Other studies verified the ability of the NS algorithm to effectively discriminate foods according to their nutritional quality [58,59]. In addition, some of the exemplary papers within this topic also suggest some improvements to the NS algorithm to also consider the presence of specific ingredients, such as nuts [60] or whole grains [61]. The comparison of the NS algorithm with other nutrient profiling systems is an issue addressed within the “Different nutrient profiling systems topic” (7.5% of the corpus). Studies focusing on this topic usually utilize a reference system to validate one or more alternative systems [62], while they often identify some discrepancies between the ratings obtained using different FOPLs [63,64].

A final class of topics is the one where the NS is not of interest “per se”, but is merely used as a tool to measure the nutritional quality of food products. Within these topics, therefore, the objective is the nutritional evaluation of specific products, despite slightly different perspectives possibly being adopted. The “Advertisements drive unhealthy food choices” (9.5%) topic focuses on the valuation of advertised products. Most of these studies observe that there is some association between the low nutritional quality of products and the advertisement discourses and strategies [65,66,67], while several studies estimated advertised products intended for children and younger generations to be of low nutritional quality [68,69,70]. The level of processing of food products and its relevance for nutritional quality is explored in the “NS and ultra-processed foods” (6.5%). Also in this case, the NS is used to assess the nutritional quality of products. In this respect, a couple of studies [71,72] found that there is no relation between the level of food processing and the NS grade (the NS was indeed devised to just communicate nutritional quality). Finally, the topic “Assessment of nutritional quality of food through NS” (8.0%) is more general in nature, mainly evaluating the nutritional quality of specific products (especially innovative ones, like in [73] or in [74]), or of whole food baskets [75] and meals [76].

## 4. Discussion

**Figure 8:**
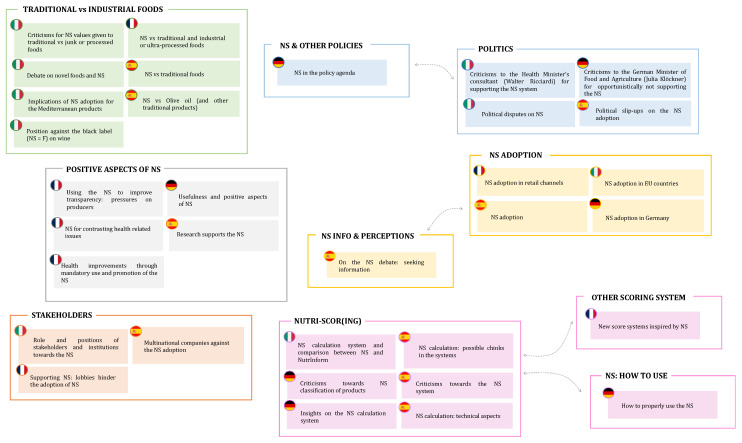
Classification of the national Twitter topics according to their content.

The results illustrated in the previous section highlight that the NS debate moves along some broad common paths in the four considered countries, but that national specificities do also exist, either in the way these paths are addressed or in the presence of specific aspects of interest. Figure 8 provides a possible classification of the identified national topics, which aims at facilitating critical discussion and considerations, without being meant to be a conclusive one.

**Figure 9:**
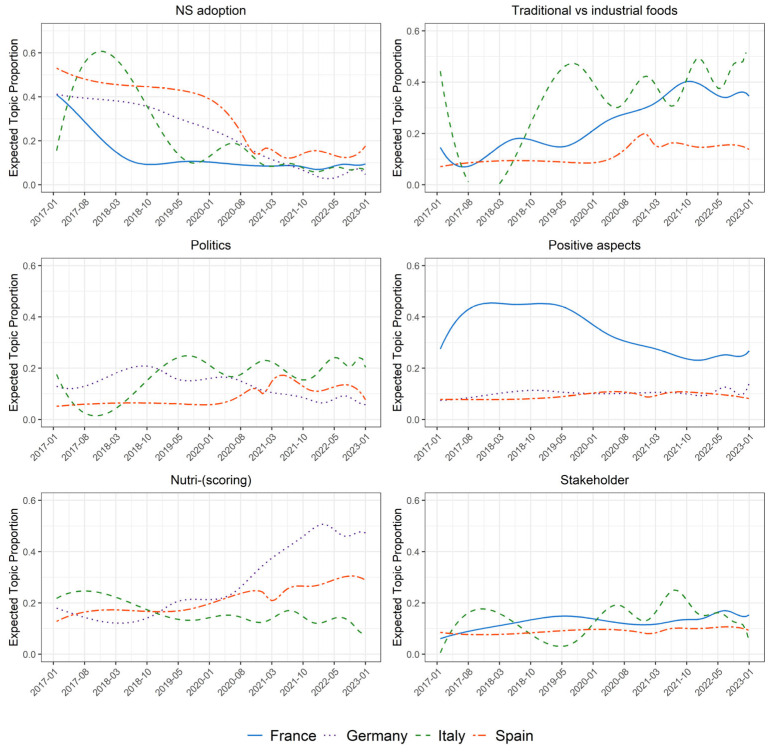
Estimated temporal trends of topic clusters by country.

In every country, people talk about the adoption of the NS in their homeland as in other EU countries, as shown in Figure 9, which reports the prevalence of the clusters defined in Figure 8 over time (obtained aggregating the individual topics’ prevalence). Discussions about “NS adoption” were in fact a hot topic when France (2017) and Belgium (2018) decided to give legal recognition to this FOPL and the EU envisaged, within the F2F strategy, a possible mandatory use of the NS on pre-packed food. Afterwards, the interest in the NS-adoption subject declined, with the exception of Spain, where the three years that elapsed between the first government proposal (in 2018) and the final NS adoption (in 2021) likely sustained the debate.

The role of national governments in the issue inevitably brings “Politics” to the fore. Apart from France, where the final NS adoption in 2017 might have somewhat settled the merely political debate, in the other three countries, discussions characterized by an intense political flavor recursively appeared. While the specific themes of these discussions clearly have a strong national component, the general sentiment transpiring from them is also quite diverse in the three contexts. In Italy, where the target of this kind of tweet is individuals supporting the NS in the national political arena and, most often, EU institutions, a strong opposition to the NS system is advocated. A negative attitude is also present in the Spanish tweets, despite the main target being the national government, especially after some of its members revealed some inconsistencies in their stance about the NS topic. Conversely, the critics of the federal government in Germany argue in the opposite direction, asking for a more active role of the government in the adoption of the NS tool.

A similar heterogeneity in approaching a common theme is found when the discussion is about “Stakeholders”. In Italy, again, various types of people linked to the food sector (e.g., professional associations, consortia, producers’ organizations) express their disagreement with the NS system, trying to prevent its adoption at the national level. On the opposite side, the discourse in France and Spain is usually directed towards a critique of large companies resisting the NS, in an attempt to press them to use the tool to promote a more transparent food system.

Transparency, indeed, is considered one of the “Positive aspects” of NS, which is claimed to allow consumers to make informed choices. Looking on the bright side is more common in countries that have already issued an NS legislation (France, Germany, and Spain), while it is rarely done in Italy. Whether the acknowledgment of the NS positive aspects by the general public is a cause or an effect of the national adoption of the system might be an interesting question to address in future research.

France is the country where the positive aspects have been stressed the most, but Figure 9 shows that they lost some importance in recent years, especially to the benefit of debates on “Traditional vs. industrial foods”. The relation between NS and traditional products mainly interests the three Mediterranean countries (France, Italy, and Spain) and, as for the specific French case, has tended to increase in the last few years. The attention to this issue is likely to be related to the strong importance in these countries of geographical indications (GIs). On the one hand, the presence of GIs has been considered an indicator of a food culture strongly based on traditions and traditional products [77]. In addition, some of the largest GIs in these countries, which are mainly related to the meat, cheese, and olive oil sectors, will likely be negatively affected by the introduction of the NS [11]. While in France and Spain, the discussion is mostly concerned with the NS classification of GI and other traditional products, the Italian debate goes further. Indeed, Italian users seem to place the NS within a broader conflict between national culinary habits and traditions and novel, foreign, and “artificial” foods that risk replacing the local food culture.

While these arguments are characterized by a strong identitarian component, criticisms of the NS are also put forward in a less ideological way, for example by looking at the potential flaws in the NS algorithm. These kinds of discussions, which are grouped in the “Nutri-scor(ing)” cluster, appear in Italy, where the main concern seems to be the comparison with the Italian-proposed nutritional label (i.e., Nutrinform), as well as in Germany and Spain, where their importance is growing. Interestingly, in the latter countries, some debates are observed that denote a good knowledge of the topic, and also its technical aspects. Specifically, issues are mentioned such as the need to account, in the nutritional evaluation of food, for the size of the portions and the composition of the whole meal, as well as the importance in limiting the use of the NS for comparing products within the same food category.

### Comparing Science and Society

**Figure 10:**
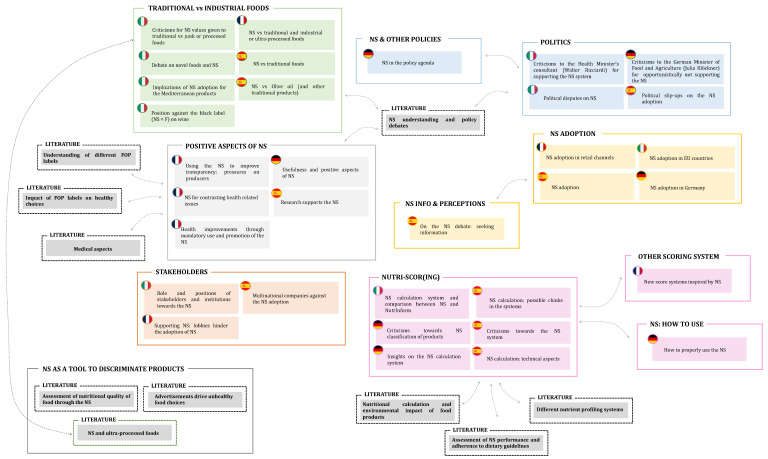
Comparison between the topics emerged from the scientific literature (grey squared; dotted line) and tweets on the NS label.

Given the diversified issues raised in the four considered countries, a clear need emerged to understand the extent to which the scientific community has addressed the aspects that stemmed from the public debate. Indeed, to decide what FOPL to adopt at the EU level, the European Commission needs to have a complete overview of the NS topic, evaluating all its technical features while considering, at the same time, the most relevant issues raised by citizens and politics. To this extent, in Figure 10, all the topics that emerged from the literature analysis are placed side by side those retrieved from tweets (Figure 8) to display in a clear way the similarities and differences between the scientific and the public debate.

As shown in Figure 10, not all the main topics discussed at a political and societal level are addressed by researchers, as some Twitter topics appear not to be related to the scientific ones. Unsurprisingly, the “NS adoption” topic finds no corresponding interest at the scientific level, as it is not a meaningful aim of scientific research. Indeed, even if most of the papers deal with NS adoption (e.g., [6,78]), describing, in different countries, how and when various FOPLs (including NS) were adopted at the European level, this is never considered the primary aim of these papers. On the contrary, extensive correspondence is found when considering the “Positive aspects” associated with NS adoption. As widely discussed in many papers (“Understanding of different FOP labels”), the NS has been strongly supported at the European level, being considered one of the easiest-to-understand FOPLs [6,79]. Providing simple information about the nutritional content of foods, and thus reducing the information asymmetry, NS seems to guide consumers towards healthier choices, as widely described in the literature topic “Impact of FOP labels” [80,81]. Choosing the healthiest products turns, inevitably, into a virtuous cycle, whereby diet-related diseases, such as obesity, renal diseases, and cancer, seem to (potentially) decrease in patients using NS [52,82], as described in the “Medical aspects” papers.

Despite these promising premises, however, the literature lacks in analysis of how this label might impact the market dynamics, both on the producers’ and consumers’ side. Twitter analysis has indeed highlighted a general reluctance of some food industries to adopt the NS (“Stakeholders”), although no matching topic was found in the literature. Indeed, only one paper [10] deals with this aspect, underlining how Italian stakeholders question the NS effectiveness on multiple levels: cognitive, normative, and political. At the political level, much attention has been paid to this issue, stressing the possible negative effect that this labeling could have on some products (or product categories). This is especially true for GI products, which cannot be easily reformulated, as is the case for the industrial ones, due to their product specification. This aspect, while much debated on the web (“Traditional vs. industrial foods”), has only been considered by a handful of articles [10,11].

The same does not apply to the relationship between ultra-processed products and NS, largely criticized by consumers and politicians. Indeed, different ultra-processed products are considered as the healthiest option (NS equal to “A” or, at least, “B”) by the NS algorithm, even if the consumption of Ultra-Processed Foods (UPFs) has been associated with low diet quality, obesity, and adverse health effects [83]. The literature partially addresses this issue, even if, in most of the cases, the NS has been used as a tool to discriminate products according to their nutritional profile rather than considering the NS as the main topic of the research (e.g., [83,84,85]). However, several authors, such as Valenzuela et al. [63] or Romero Ferreiro et al. [86], have addressed this issue, highlighting some discrepancies within the two labeling systems. The calculation mechanism behind the NS has been, in fact, strongly criticized by Twitter users in several countries, as suggested by the topics reported in the “Nutri-Scor(ing)” cluster. From the scientific side, some improvements to the algorithm have been proposed, such as including nuts [60] or whole grains [61] as positive elements, to better follow the path of healthy eating.

In line with this aspect, a general disappointment also emerges when considering the NS evaluation of the Mediterranean diet products (“Assessment of NS performance and adherence with dietary guidelines”*)*. According to some Twitter users, the algorithm, in fact, seemed to “damage” some of these products. However, as explained by Vlassopoulos et al. [54], the NS is perfectly in line with the Mediterranean diet, as products of animal origin, evaluated negatively by the NS, are also considered products to be consumed with limitations from the Mediterranean diet precepts. This opens up an important food for thought. In most scientific papers, in fact, products with NS “D” or “E” are generally considered as “unhealthy” products, while the NS guidelines (at least in the latest version) define sugars, fatty acids, calories, and salt as elements and ingredients “to be limited in consumption”, which does not imply a totally negative evaluation of the product itself, but simply an indication of use. In light of what emerged from the analysis of the tweets and of the literature, it is, however, clear that this difference is not clearly understood by consumers and, probably, should be better clarified to make the label truly effective. Indeed, as discussed by Stiletto and Trestini [11], in countries unfamiliar with the NS, such as Italy, consumers with a low awareness of the label evaluate it as an element of product quality, regardless of the score assigned to it. This means that for the NS to be effective, supplementary information on what the NS is and how it works should be provided, using words and systems understandable in all EU countries. At the same time, studies aimed at determining the effectiveness of NSs in guiding consumers’ food choices should be conducted in all EU countries, considering that familiarity with the label is one of the main factors affecting its efficacy [5]

This study is subject to some limitations. First, it should be considered that a Twitter text is quite short, potentially affecting the ability to express viewpoints in a clear way. Second, we used Twitter as the only data source to ascertain public opinion on the NS, while other social media or grey literature could also be potentially used to reach this objective. In addition, it must be kept in mind that Twitter users (as in the case of social media in general) might not be representative of the whole population [19]. In this respect, further studies will be useful to extend the scope of this analysis to segments of the population that are at risk of being underrepresented in a social media context. Finally, this study lacks a comparison between the Twitter and literature trends. However, this limitation, which is essentially due to the novelty of the NS topic, will be easily addressed in a few years, when a wider series of published scientific material on NS will be available.

## 5. Conclusions

Our study stressed that the NS debate is relevant and heterogeneous across Europe. At the EU institutional level, NS benefits from a quite large amount of support, being considered the most effective FOPL in guiding consumers’ choices towards healthier food products. This view is substantiated by several scientific studies, which proved that NS actually promotes healthier food choices, while performing better than other FOPLs.

Despite this evidence, however, consumers and policy makers all over Europe have pointed out some critical issues related to the use of the label that, if not adequately addressed, could undermine its effectiveness in the long run. Among others, the (potential) negative effect of NS on some products (such as traditional products) is the most mentioned one, especially in Mediterranean countries. In addition, some inconsistencies in the calculation system are brought to attention, as well as some criticalities concerning the correct interpretation of the label.

To help settle this debate and address the concerns raised by consumers and stakeholders, further research is needed. Specifically, new literature on the topic can play a twofold role, based on the results that will emerge from future studies. On the one hand, scientific research outcomes, if properly communicated, can reassure the public opinion on issues and concerns that turn out to be unfounded. On the other hand, if the existence of some flaws in the NS is actually proved, tailored research can serve as the basis on which to improve the NS tool. With respect to the latter aspect, this has already happened, for example, in the case of olive oil and nuts, whose original misclassification led to a revision in the NS algorithm.

Widening the NS research to explore the concerns and issues raised by society has therefore the potential to facilitate policy decisions. In fact, while it would be unreasonable to imagine the removal of any critique, having a complete vision of the NS topic derived from research might allow the legislator to justify the final decision (whichever it will be) on a more solid ground.

However, it should also be acknowledged that scientific research alone might not be enough. Our analysis showed that several criticisms of the NS system stem from a misinterpretation of the label. This evidence suggests that any policy decision on the issue should be accompanied by communication activities aimed at informing consumers and stakeholders about what the NS is, how it works, and how to properly use it. Otherwise, paradoxically, a tool created to reduce information asymmetry seems instead to be a slave to it. For example, explaining that NS suggests the recommended consumption dose of a product and does not classify it as “healthy” or “unhealthy” would contribute to alleviating some of the distrust towards this label. At the same time, creating information campaigns related to the correct use of the label, so that it is also useful to those consumers who are unfamiliar with the NS and may therefore misinterpret it, might be the best way to increase the label’s effectiveness and reach its intended outcome, namely reducing the rate of obesity and overweight in Europe.

Further research should analyze the impact of the Nutri-Score on the market dynamics, from both a producers’ and consumers’ side, especially considering Traditional Foods, such as Geographical Indications. In addition, as the NS topic is not equally investigated in all the European Countries (although it is a EU policy), NS consumers’ understanding should be investigated in all the European countries, especially in those with low familiarity with the label.

## Figures and Tables

**Table 1:** Number of geolocated tweets in the considered period of analysis (2017–2023) posted in non-native languages.

Country	Number of Tweets	Tweets in the Native Language	Share of Native Tweets (%)
France	568	496	87.3
Germany	167	139	83.2
Italy	229	167	72.9
Spain	533	466	87.4
Total	1497	1268	84.7

**Table 2:** Number of original tweets about NS.

Country	Number of Tweets	Tweets/(Year × 1000 Users)
France	26,535	440
Germany	11,431	250
Italy	8981	310
Spain	18,776	360
Total	65,723	350

## Data Availability

This study followed Twitter’s Developer’s Terms and Policy (available at: https://developer.twitter.com/en/developer-terms/policy, accessed on 31 January 2023), which has restrictions on redistributing Twitter content to third parties.

## Appendix A

**Table A1:** Topics identified in the Twitter corpus.

Topic	Most Typical Terms	Prevalence	Exemplary Tweets (Native Language)	Exemplary Tweets (English Translation)
ITALY				
**T1** Debate on novel foods and NS	eat nothing good sense read grasshoppers worms synthetic dish want	11.1%	L’UE sta disintegrando il nostro vivere millenario, vuol farci mangiare insetti che contengono questi parassiti e mette il NUTRISCORE più alto su coca cola che non il Parmigiano Reggiano!	The EU is disintegrating our millenary life, it wants us to eat insects that contain these parasites and puts the highest NUTRISCORE on coca cola than Parmigiano Reggiano!
			IL PARADOSSO UE: GLI INSETTI SÌ, IL PARMIGIANO NO! Prima con il Nutriscore l’UE mette in discussione i nostri prodotti bandiera (Parmigiano, olio d’oliva, ecc) e poi dà l’ok a farci mangiare insetti e larve? A voi i commenti…	THE EU PARADOX: INSECTS YES, PARMIGIANO NO! First with the Nutriscore, the EU questions our flagship products (Parmesan, olive oil, etc) and then gives the ok to let us eat insects and larvae? To you the comments…
			L’Europa non ci costringe a mangiare insetti e bere vino annacquato. Il Nutriscore è uno strumento di valutazione sulla salubrità del cibo, nessuno ci obbliga a seguirlo. Mangiamo già insetti, una minima parte è tollerata dalla legge perché facentes parte del processo produttivo	Europe does not force us to eat insects and drink watered wine. The Nutriscore is an assessment tool on the wholesomeness of food, nobody forces us to follow it. We already eat insects. A small part is tolerated by law because it is part of the production process.
**T2** Role and position of Stakeholders and Institutions towards NS	Patuanelli legal protection president supply chain Confargicoltura antitrust PGI Federalimentare future interview	13.9%	Continua il dibattito sul Nutriscore, Asti Agricoltura: “Confidiamo nel Governo affinché tuteli l’intera filiera agroalimentare italiana	The debate on Nutriscore is still going, Asti Agricoltura: “We trust the Government to protect the entire Italian agri-food chain
			@origin_italia ha incontrato il Ministro @SPatuanelli: al centro dell’incontro la Riforma del sistema delle #DOP #IGP, il #Nutriscore, i contratti di filiera per il #PNRR e la nuova #PAC	@origin_italia met the Minister @SPatuanelli: the Reform of the #PDO #IGP system, the #Nutriscore, the supply chain contracts for the #PNRR and the new #CAP were at the center of the meeting
			#Draghi sul #Nutriscore alla Camera dei Deputati: “Il Governo è totalmente consapevole della gravità che l’introduzione del Nutriscore può costituire per la nostra filiera produttiva agroalimentare e pienamente impegnato nella sua tutela”	#Draghi on the #Nutriscore in the Chamber of Deputies: “The Government is fully aware of the gravity that the introduction of the Nutriscore can represent for our agri-food production chain and fully committed to its protection”
**T3** Political disputes on NS	Italians Meloni politics g*reen* Lollobrigida approved agenda very *sound* hands	10.2%	Giorgia Meloni, porcheria Nutri- Score: “Von der Leyen, dovrai passare sopra il mio corpo”	Giorgia Meloni, Nutri-Score filth: “Von der Leyen, you will have to go over my body”
			Vittorio Feltri distrugge l’Europa sulla certificazione dei cibi: “Imbecillità totale”. Non sono imbecilli in UE, perseguono il loro obiettivo primario..rendere l’Italia una nazione povera, sotto controllo dell’UE…	Vittorio Feltri destroys Europe on food certification: “Total imbecility”. They are not imbeciles in the EU, they pursue their primary goal.. to make Italy a poor nation, under the EU control…
			Ahhh le menzogne traditori sono talmente divertenti per i soliti pecoroni!I PiDioti hanno approvato il Nutriscore in Europa che ammazza i nostri prodotti e le nostre imprese! Hanno approvato l’agenda green che ammazza la nostra industria automobilistica! Il PD distrugge l’Italia	Ahhh the treacherous lies are so much fun for the usual idiots! The (PD)idiots have approved the Nutriscore in Europe which kills our products and our businesses! They approved the green agenda that is killing our car industry! The PD destroys Italy
**T4** Implications of NS adoption for Mediterranean products	*Made* *Italy* food Italian Mediterranean attack risk *export* synthetic threat	9.3%	Nutriscore, ora il Made in Italy trema davvero	Nutriscore, now Made in Italy is really trembling
			Made in Italy, Coldiretti: il via libera all’etichetta Nutriscore che rischia di espandersi a livello globale mette in pericolo il record di 46,1 miliardi di esportazioni agroalimentari tricolori del 2020	Made in Italy, Coldiretti: green light for the Nutriscore label which risks expanding globally endangers the record of 46.1 billion in Italian agri-food exports in 2020
			#G20, Coldiretti: omaggiare i grandi della Terra con vino o olio non è solo un’importante azione di promozione del cibo Made in Italy all’estero ma anche un preciso segnale politico a difesa della dieta mediterranea sotto attacco del Nutriscore e delle etichette allarmistiche	#G20, Coldiretti: paying homage to the greats of the Earth with wine or oil is not only an important action to promote Made in Italy food abroad but also a precise political signal in defense of the Mediterranean diet under attack by Nutriscore and labels alarmist
**T5** NS adoption in EU Countries	Italy label Germany same *Fratelli* adopted Belgium category against adopt	9.5%	Belgio: #Carrefour e #Danone adottano il #NutriScore. Arriverà prima su app e web, ed entro il 2020 sulle confezioni	Belgium: #Carrefour and #Danone adopt the #NutriScore. It will arrive first on the app and web, and by 2020 on packaging.
			Nutri-Score: anche la Spagna adotta l’etichetta a semaforo francese	Nutri-Score: Spain also adopts the French traffic light label
			Il Nutri-Score arriva anche in Italia! L’etichetta a semaforo su un prodotto Sojasun comprato da #Lidl #NutriScore #etichettaSemaforo #nutrizione #Sojasun	The Nutri-Score also arrives in Italy! The traffic light label on a Sojasun product bought from #Lidl #NutriScore #labelSemaforo #nutrizione #Sojasun
**T6** Position against the black label (NS = F) on wine	understanded black nobody to understand beverages indeed never still cancer *propaganda*	13.5%	La @Federcuochi risponde con fermezza NO all’ennesima folle proposta degli ideatori del Nutriscore, che vorrebbero etichettare con una F nera tutte le bevande contenenti anche una minima percentuale alcolica.	The @Federcuochi firmly replies NO to the umpteenth crazy proposal from the creators of Nutriscore, who would like to label all drinks containing even a minimal percentage of alcohol with a black F.
			“Stupore e sconcerto” per il tentativo di applicare il #Nutriscore a vino e a bevande alcoliche, attribuendo a esse la lettera F di colore nero. Le dichiarazioni di @sweetlemongal e #AlbieraAntinori che esprimono la contrarietà di #Federvini e del comparto	“Amaze and bewilderment” for the attempt to apply the #Nutriscore to wine and alcoholic beverages, attributing to them the black letter F. The statements by @sweetlemongal and #AlbieraAntinori expressing the opposition of #Federvini and the sector
			Zottis (Pd): “#alcolici, nessun bollino nero sulle bottiglie, il vino non è cancerogeno. Vince la chiarezza, sconfitto l’allarmismo” #Nutriscore @ZottisFrancesca	Zottis (Pd): “#alcoholics, no black label on the bottles, wine is not carcinogenic. Clarity wins, scaremongering defeated” #Nutriscore @ZottisFrancesca”
**T7** NS calculation system and comparison between NS and Nutrinform	consumers nutritional consumer Nutrinform battery information values choices correct algorithm alternative	13.2%	Il #Nutriscore si basa su un algoritmo che classifica l’alimento in base a zuccheri grassi e sale e non tiene conto dei processi di trasformazione del prodotto (una lasagna confezionata risulta più salutare di un cucchiaino di miele) penalizzando #madeinitaly e #dietamediterranea	The #Nutriscore is based on an algorithm that classifies the food according to fat sugars and salt and does not take into account the transformation processes of the product (a packaged lasagna is healthier than a teaspoon of honey) penalizing #madeinitaly and #Mediterranean diet
			Il Nutri-Score utilizza un algoritmo che tiene conto del contenuto di componenti negativi (energia, grassi saturi, zucchero e sodio) e positivi (fibra, frutta/verdura/oli e, alcune volte, proteine) in 100 g di prodotto	The Nutri-Score uses an algorithm that takes into account the content of negative (energy, saturated fat, sugar and sodium) and positive (fiber, fruit/vegetables/oils and, sometimes, protein) components in 100 g of product
			#Nutriscore crea una dipendenza nel consumatore che deve accettare le valutazioni dell’algoritmo senza comprenderne ragioni. #Nutrinform informa, senza interpretare, fornendo gli elementi per scegliere consapevolmente.	#Nutriscore creates an addiction in the consumer who must accept the evaluations of the algorithm without understanding reasons. #Nutrinform informs, without interpreting, providing the elements to make an informed choice.
**T8** Criticism of the Health Minister’s consultant (Walter Ricciardi) for supporting the NS system	Italians Ricciardi in favor Speranza penalize damage scientists signed Walter consultant	10.6%	Ricciardi a favore del Nutriscore, scoppia il caso nel Governo. Salvini: “Si dimetta” Tutti contro Ricciardi, ma è uno scienziato (uno dei 280 firmatari dell’appello) e difende la salute, non gli interessi economici delle aziende.	Ricciardi in favor of the Nutriscore, the case breaks out in the Government. Salvini: “He should resign” All against Ricciardi, but he is a scientist (one of the 280 signatories of the petition) and defends health, not the economic interests of companies.
			… IL NOSTRO È L’UNICO PAESE AL MONDO GOVERNATO DAI SUOI NEMICI. RICCIARDI SI DIMETTA!	… OURS IS THE ONLY COUNTRY IN THE WORLD GOVERNED BY ITS OWN ENEMIES. RICCIARDI MUST RESIGN!
			Walter Ricciardi, super consulente di Speranza, si è di fatto schierato a favore dell’introduzione in Europa del #Nutriscore, il sistema di etichettatura fortemente voluto dai francesi che penalizza i prodotti italiani	Walter Ricciardi, super consultant of Speranza, has in fact sided in favor of the introduction in Europe of the #Nutriscore, the labeling system strongly desired by the French which penalizes Italian products
**T9** Criticism for NS values given to traditional vs. junk or processed foods	(olive) oil Parmigiano olive red ham coca green cola Reggiano fries	8.6%	Secondo il #Nutriscore voluto dall’UE, l’olio extra vergine di oliva merita il bollino rosso e la Coca Cola zero il semaforo verde… no comment.	According to the #Nutriscore, supported by the EU, extra virgin olive oil deserves the red label and Coca Cola zero the green light… no comment.
			Grazie al Nutri-score avremo una Mozzarella di Bufala Campana DOP, lavorata a mano, segnalata come più pericolosa per la salute rispetto ad una bistecca di soia estrusa a macchina ed aromatizzata con insaporiti chimici!	Thanks to the Nutri-score we will have a Mozzarella di Bufala Campana PDO, hand-crafted, considered as more dangerous to health than a machine-extruded soybean steak flavored with chemical flavorings!
			In #Francia l’hamburger ai fast food risulta più “sano” del prosciutto di Parma Dop. E lo stesso vale per l’olio extravergine di oliva e il parmigiano. Ecco svelato a cosa serve il #Nutriscore: uccidere il #MadeinItaly	In #France, fast food hamburgers are healthier than Prosciutto di Parma PDO. And the same goes for extra virgin olive oil and Parmigiano Reggiano PDO. Here’s what the #Nutriscore is for: killing #MadeinItaly
FRANCE				
**T1** Health improvements through mandatory use and promotion of the NS	Santè food mandatory information Publique make European choose industry advertising	13.9%	Le Nutri-Score obligatoire dans les publicités des aliments. Les annonceurs pourront cependant y déroger moyennant une contribution affectée à l’Agence nationale de santé publique On rend les gens malades, mais on contribue a les soigner…	Nutri-Score (will be) mandatory in food advertisements. Advertisers will however be able to derogate from it by means of a contribution allocated to the National Public Health Agency We make people sick, but we help to treat them…
			L’Assemblée nationale a rejeté dimanche un amendement visant à rendre obligatoire dans les publicités audiovisuelles le Nutri-Score, qui indique les vertus alimentaires d’un produit.	The National Assembly on Sunday rejected an amendment aimed at making the Nutri-Score mandatory in audiovisual advertisements, which indicates the nutritional virtues of a product.
			📄 Communiqué de presse | Santé publique France lance la première campagne nationale pour faire connaître le #NutriScore auprès des consommateurs	📄 Press release | Santé publique France launches the first national campaign to promote the #NutriScore to consumers
**T2** Supporting NS: lobbies hinder the adoption of NS	how to be little other lobby good been Europe best European	13.2%	Face aux lobbys, 36 associations de professionnels de santé (nutritionnistes, diabétologues, pédiatres, cancérologues, cardiologues, acteurs de santé publique…), consommateurs et patients et ONGs appellent à signer une pétition pour défendre #NutriScore	Faced with lobbies, 36 associations of health professionals (nutritionists, diabetologists, pediatricians, oncologists, cardiologists, public health actors, etc.), consumers and patients and NGOs are calling to sign a petition to defend #NutriScore
			Pour faire changer les choses et rendre obligatoire le #NutriScore au niveau européen, une initiative citoyenne européenne a été lancée pour lutter contre la #malbouffe. Pour la santé publique contre les lobbys qui s’y opposent, SIGNEZ LA PETITION http://pronutriscore.org	To change things and make #NutriScore compulsory at European level, a European citizens’ initiative has been launched to fight against #junk food. For public health against the lobbies that oppose it, SIGN THE PETITION http://pronutriscore.org
			Merci @isabellesaporta mais la bataille n’est pas finie. De puissantes multinationales continuer a refuser #Nutriscore: Kelloggs, Ferrero, Mars, Unilever, Mondelez, Coca, Pepsi… Pour leur forcer la main il faut signer en masse la pétition européenne http://pronutriscore.org	Thanks @isabellesaporta but the battle is not over. Powerful multinationals continue to refuse #Nutriscore: Kelloggs, Ferrero, Mars, Unilever, Mondelez, Coca, Pepsi… To force their hand, you have to sign the European petition en masse http://pronutriscore.org
**T3** New score systems inspired by the NS	organic have by impact recipe idea fruits effect given rated	15.6%	Visite ce matin de l’entreprise Jacquet-Brossard l’occasion de parler économie sociale et circulaire, filières bio, nutriscore, insertion par l’emploi, coopérative @Limagrain @CoopdeFrance @lamontagne_fr @F3Auvergne @FBAuvergne @RCFPuydeDome	Visit this morning of the Jacquet-Brossard company the opportunity to talk about social and circular economy, organic sectors, nutriscore, integration through employment, cooperative @Limagrain @CoopdeFrance @lamontagne_fr @F3Auvergne @FBAuvergne @RCFPuydeDome
			Après le NutriScore pour l’alimentation, voilà le CyberScore pour la sécurité des sites. A lire sur @Numerama 📃 “Le texte doit déboucher par la mise en place d’une certification de cybersécurité des plateformes numériques destinée au grand public	After the NutriScore for food, here is the CyberScore for site security. To read on @Numerama 📃 “The text must lead to the establishment of a cybersecurity certification for digital platforms intended for the general public
			Et si un “Nutriscore” de la responsabilité sociale des entreprises voyait le jour, en mesurant une quinzaine d’indicateurs transversaux et structurants sur quatre piliers: l’impact social et environnemental, le partage des richesses et du pouvoir?	What if a “Nutriscore” of corporate social responsibility were created, measuring fifteen cross-cutting and structuring indicators on four pillars: social and environmental impact, sharing of wealth and power?
**T4** NS vs. traditional and industrial or ultra-processed foods	good same good note cheese processed score fat few nothing	28.8%	Les producteurs de #Roquefort demandent à être exemptés du #NutriScore. Le fromage au lait de brebis de l’#Aveyron est mal classé, en raison de ses taux de sel et d’acide gras saturé	#Roquefort producers ask to be exempted from #NutriScore. Sheep’s milk cheese from #Aveyron is poorly classified, due to its salt and saturated fatty acid levels
			Steak 100% pur boeuf score C Steak de soja score A (ultra transformé a base d’eau, huile, protéine en poudre, et autres additifs)… Depuis que j’ai vu ça j’ignore le nutriscore	Steak 100% pure beef score C Score A soybean steak (ultra-processed with water, oil, protein powder, and other additives)… Since I saw that I ignore the nutriscore
			Tout les fromages (la plupart) ont un Nutriscore degueulasse. Forcément, le fromage c’est quasiment du gras. Et alors? Les gens savent se qu’ils achètent quand ils prennent du fromage. Donc j’ai envie de dire: on s’en fout du nutriscore	All cheeses (most) have a disgusting Nutriscore. Inevitably, cheese is almost fat. So what? People know what they’re buying when they get cheese. So I want to say: who cares about the nutriscore
**T5** NS adoption in retail chains	do label nutritional logo already used calculation know study engaged	10.4%	Devant Marisol Touraine et la CLCV, Intermarché, Leclerc, Auchan et Fleury Michon s’engagent à utiliser Nutriscore	In front of Marisol Touraine and the CLCV, Intermarché, Leclerc, Auchan and Fleury Michon commit to using Nutriscore
			Un nouveau logo nutritionnel arrive sur les aliments dès avril: le NutriScore	A new nutritional logo is coming to food in April: the Nutri Score
			La nouvelle étiquette #Nutriscore dans nos rayons en avril 🛒 #alimentation #nutrition #sante #food #packaging #info	The new #Nutriscore label on our shelves in April 🛒 #food #nutrition #health #food #packaging #info
**T6** Using the NS to improve transparency: pressures on producers	consumer industrial all again labelled choice transparent question point something	10.4%	Nutriscore: Les marques qui l utilisent ont choisi la transparence vis à vis des consommateurs. D autres n ont pas voulu, voire lutté contre… Privilégiez les marques qui l ont adopté ! #santé #prévention #nutriscore @sfsp @santeprevention @MinSoliSante @HercbergS	Nutriscore: The brands that use it have chosen transparency for consumers. Others did not want, even fought against… Choose brands that have adopted it! #health #prevention #nutriscore @sfsp @santeprevention @MinSoliSante @HercbergS
			L’application a le succès qu’elle mérite! Si les consommateurs l’utilisent c’est que ce sont les industriels et les distributeurs qui ne jouent pas la transparence sur leurs produits! A quand la vignette Nutri Score sur tous les emballages ? #malbouffe	The application has the success it deserves! If consumers use it, it is because manufacturers and distributors are not transparent about their products! When will the Nutri Score label be on all packaging? #junk food
			#Nutriscore: « La pression des consommateurs peut faire plier les industriels ».Tribune dans le Parisien Dimanche	#Nutriscore: “Consumer pressure can make manufacturers bend”. Tribune in the Parisian Sunday
**T7** NS for contrasting health-related issues	food nutritional nutrition Yuka French label according to app public interest	7.7%	Le Nutri-score se révèle le plus efficace pour mesurer la qualité nutritionnelle des aliments. #alimentation #nutrition @veillesante @Anses_frm@AlimentSante @IsabelMalsang @leQdM	The Nutri-score is the most effective way to measure the nutritional quality of foods. #alimentation #nutrition @veillesante @Anses_frm@AlimentSante @IsabelMalsang @leQdM
			Nutri-Score: attention, les aliments mal notés augmentent les risques de cancer	Nutri-Score: attention, poorly rated foods increase the risk of cancer
			Le Nutri-Score a été choisi fin octobre 2017 par la France pour mieux informer les consommateurs sur la qualité nutritionnelle des aliments. Selon une étude, les aliments mal notés par le #NutriScore augmentent le risque de cancer	The Nutri-Score was chosen at the end of October 2017 by France to better inform consumers about the nutritional quality of food. According to a study, foods with low #NutriScore scores increase the risk of cancer
GERMANY				
**T1** Criticisms of the German Minister of Food and Agriculture (Julia Klöckner) for opportunistically not supporting the NS	Klöckner food traffic light voluntarily study Julia Mrs finally industrial food industry Minister	12.2%	Das Tanzmarichen der Lobbyisten Kennzeichnung Nutri-Score: Wie Ministerin Klöckner die Lebensmittelampel behindert	The lobbyists’ dance Marking Nutri-Score: How Minister Klöckner obstructs the food traffic light
			@foodwatch_de berichtet: Im Streit um die Nährwertkennzeichnung von Lebensmitteln ließ das Ernährungsministerium von Julia Klöckner offenbar eine wissenschaftliche Studie stark umschreiben, die dem Nutri-Score ein gutes Zeugnis ausstellt	@foodwatch_en reports: In the dispute over nutritional labeling of food, Julia Klöckner Ministry of Food apparently had a scientific study heavily rewritten that gives the Nutri-Score a good mark
			@JuliaKloeckner verheimlicht uns eine Studie zur Lebensmittelampel, die dem #Nutriscore offenbar ein gutes Zeugnis ausstellt, und veröffentlicht Monate später nur eine überarbeitete Fassung. Schluss mit der Geheimniskrämerei! Her mit der Ampel-Studie	@JuliaKloeckner hides from us a study on the food traffic light, which apparently gives the #Nutriscore good marks, and only publishes a revised version months later. No more secrecy! Bring on the traffic light study
**T2** NS adoption in Germany	nutrition label Germany introduction logo food labeling model France fight stigma opinion poll	17.0%	#nutriscore ist eine französische Erfindung, nicht belgisch:) aber in Frankreich, Belgien, Spanien, Polen, Portugal, Litauen, der Schweiz verwendet: es bewegt sich in Europa!	#nutriscore is a French invention, not Belgian:) but used in France, Belgium, Spain, Poland, Portugal, Lithuania, Switzerland: it’s moving in Europe!
			Deutschland sucht sein #Nährwert-Logo. Der klare Favorit des @vzbv: #NutriScore. Die farbliche #Nährwertkennzeichnung erleichtert es Verbrauchern nachweislich gesündere Alternativen auf einen Blick zu erkennen. #ProNutriScore	Germany is looking for its #nutritional value logo. The clear favorite of @vzbv: #NutriScore. The colored #nutrition labeling makes it easier for consumers to identify healthier alternatives at the first glance. #ProNutriScore
			Lebensmittelkennzeichnung: Landgericht Hamburg stoppt Nutri-Score vorübergehend: Hamburg—Das Landgericht Hamburg hat eine einstweilige Verfügung gegen die Kennzeichnung von Iglo-Verpackungen mit dem Nutriscore… #NutriScore #BMEL #Klckner #BLL #iglo	Food labeling: Hamburg district court temporarily stops Nutri-Score: Hamburg—The Hamburg district court has issued an injunction against the labeling of Iglo packaging with the NutriScore #NutriScore #BMEL #Klckner #BLL #iglo
**T3** Criticisms towards NS classification of products	just fries know bad find on it compare vegan seen cheese	23.8%	HAB GESEHEN DAS MEINE LIEBLINGS HARFER KEKSE NUTRI SCORE E HABEN UND ICH WAR SO ERSCHÜTTERT??? ich dachte die wären eig ganz gesund was soll die scheiße	I SAW MY FAVORITE HARFER BISCUITS HAVE NUTRI SCORE E AND I WAS SO SHOCKED??? I thought they were really healthy, what the heck
			Käse einen Nutri-Score zwischen C und D (hab echt noch nie welchen mit A gesehen) und Nudeln ALLESAMT (egal, ob helle Weizen-, Dinkelvollkorn- oder Kichererbsennudeln) ein A (:	Cheese has a Nutri-Score between C and D (I’ve really never seen one with an A) and pasta ALL (regardless of whether light wheat, whole meal spelled or chickpea pasta) an A (:
			Die Pommes und das Toastbrot haben Nutriscore A. Und die Pommes sind sogar vegan!! Alles total gesund. Lasst euch nix erzählen!!!!!!	The fries and toast are Nutriscore A. And the fries are even vegan!! Everything totally healthy. Don’t let me tell you anything!!!!!!
**T4** Usefulness and positive aspects of NS	were people help best shopping right years see read understand	10.5%	Mol ofgesinn vun der willkürlecher Bewertung a dem Choixass dat alles aanescht wei transparent an informativ. Dat kann een normale Konsument net novollzeien, an d’Informatioun iwer den Nutriscore ass net mei einfach ze verstoen wei d’lëscht vun den Inhaltsstoffer	From the point of view of the arbitrary evaluation on the Choixass, everything is transparent and informative. A normal consumer can’t accept that the information about the Nutriscore is simply not available because the ingredients are not known
			Nienamd! Und genau deshalb sind vereinfachte Kennzeichnungen wie NutriScore und Co. ja auch eine gute Idee. Weil sie dem Verbraucher auf einen Blick einen Hinweis geben, was er da kauft. Ohne dass er zuvor Oecotrophologie studieren muss.	No man! And that’s exactly why simplified labels like NutriScore and Co. are a good idea. Because they give the consumer an indication of what they are buying at a glance. Without having to study ecotrophology beforehand.
			Genuss und Verantwortung @RenateKuenast - bei vielen Lebensmittel wird suggeriert sie wären Grundnahrungsmittel—Verbrauchernnen haben das Recht zu erfahren, was drinsteckt #NutriScore—viel Beifall auf #zeitauftrag @ZEITvst	Enjoyment and responsibility @RenateKuenast—with many foods it is suggested that they are staple foods—consumers have the right to know what’s in them #NutriScore—much applause on #zeitauftrag” @ZEITvst
**T5** NS in the policy agenda	Nestlé come our year theme November Federal Council companies free Corona	11.8%	Die Politik hat die Aufgabe esellschaftliche Missstände zu regeln. Was für die @cducsubt aktuell keiner Regelung bedarf? -126.000 Küken im Schredder jeden Tag -Kastenstände +17 Jahre -Nutriscore and Tierhaltungslabel nur freiwillig -Bodenversalzung durch Gülle -Profit vor Ethik	Politics has the task of regulating social ills. What for the @cducsubt currently needs no regulation? -126,000 chicks in the shredder every day -Cage stalls +17 years -Nutriscore and livestock label only voluntary -Salting of soil by manure -Profit before ethics
			November 2020: Das ändert sich in Deutschland, Neue Quarantäne-Regelungen, Berliner Flughafen BER, der Nutri-Score und Änderungen bei der Kfz-Steuer—es gib neue Gesetze und Regelungen im November 2020 in Deutschland	November 2020: This changes in Germany, New quarantine regulations, Berlin Airport BER, the Nutri-Score and changes in the motor vehicle tax—there are new laws and regulations in November 2020 in Germany
			Ca. 150k Tote werden jährlich durch falsche Ernährung verursacht und den Nutri-Score verhindert unsere Regierung mit großer Leidenschaft. Wir tragen die Entscheidungen i.S. Corona ja komplett mit, aber beim Thema Ernährung fehlt diese Entschlossenheit.:(	About 150k deaths are caused annually by improper nutrition and the Nutri-Score is prevented by our government with great passion. We support the decisions about Coronavirus completely, but on the subject of nutrition this determination is missing.:(
**T6** Insights into the NS calculation system	goes correct healthy declarations nutrition interest opinion meet unhealthy week	9.9%	Verwässern bedeutet verbessern?—Wie mit dem Nutri-Score getrickst wird sup.—Ein Versehen? Oder ein Fehler im System? #Ernährungsexperten wundern sich über die system—#DetlefBrendel #NutriScore #PlassenVerlag #SchlussMitEssverboten	Dilute means improve?—How to cheat with the Nutri-Score for improving it—A mistake? Or a bug in the system? #Nutrition experts wonder about the system…—#DetlefBrendel #NutriScore #PlassenVerlag #SchlossMitEssbanen
			Vorschlag: den intuitiv und schnell verständlichen Nutri-Score (der zudem in Studien getestet ist und sich etwa in Frankreich bewährt hat) auf die Vorderseite, detaillierte Angaben für alle, die es genauer wissen oder angeben möchten, auf die Rückseite	Suggestion: the intuitive and easy-to-understand Nutri-Score (which has also been tested in studies and has been proven in France, for example) on the front, detailed information for anyone who wants to know more or want to specify it on the back.
			Ein führender französischer Ernährungsforscher zum leidigen NUTRISCORE: “…the bases and tenets of this algorithm are debatable and, indeed, much debated... the fat section of the Nutriscore algorithm is wrong...It is outdated and has no basis…” Genau!	A leading French nutrition researcher on the vexed NUTRISCORE: “…the bases and tenets of this algorithm are debatable and, indeed, much debated.... the fat section of the Nutriscore algorithm is wrong...It is outdated and has no basis…” Exactly!
**T7** How to properly use the NS	product sugar few make fat actual salt unfortunately example within	14.7%	Mit dem Nutri-Score lassen sich Produkte innerhalb einer Kategorie miteinander vergleichen. Beispielsweise Pizza mit Pizza: Eine Pizza mit „B“ hat eine günstigere Nährstoffzusammensetzung als eine Pizza mit „D“. Ein Vergleich von Pizza mit TK-Gemüse ist dagegen nicht sinnvoll	The Nutri-Score can be used to compare products within a category. For example, pizza with pizza: A pizza with “B” has a more favorable nutrient composition than a pizza with “D”. A comparison of pizza with frozen vegetables, on the other hand, is not meaningful
			Der Nutri-Score nimmt eine Bewertung der Produkte ausschließlich anhand von Nährwerten vor. Die besonderen Anforderungen an Bio-Produkte finden keine Berücksichtigung und ein Vergleich mit konventionellen Produkten ist nur unzureichend möglich	The Nutri-Score evaluates products solely on the basis of nutritional values. The special requirements for organic products are not taken into account and a comparison with conventional products is only barely possible
			wenig, wenn sie einen hohen Gehalt an gesättigten Fettsäuren “kleinrechnen” können in einem einzigen Score. Der Körper verwertet die unterschiedlichen Nährstoffkomplexe und Vitamine/Spurenelemente ja nicht auf zusammengefasste Weise wie ein Nutriscore das suggeriert	It’s not enough, if they can “minimize” a high content of saturated fatty acids in a single score. The body does not utilize the different nutrient complexes and vitamins/trace elements in a combined way as a nutriscore suggests
SPAIN				
**T1** On the NS debate: seeking information	diet sector funny company cheese Mediterranean in favor of great important asks	10.5%	El etiquetado NutriScore del PSOE discrimina la dieta mediterránea y podría llegar a afectar a importantes sectores de la empresa murciana, como las conservas, cárnicas y almazaras	The PSOE’s NutriScore label discriminates against the Mediterranean diet and could affect important sectors of the Murcian company, such as preserves, meat and oil mills
			Para conocer mejor las bases científicas y las respuestas a críticas fundadas o no sobre Nutri-Score, os aconsejo escuchar la conferencia en el webinar organizada por La Vocalía de Alimentación del Consejo General de Colegios Farmacéuticos de España	To better understand the scientific bases and the responses to criticisms that are founded or not on Nutri-Score, I advise you to listen to the conference in the webinar organized by the Food Committee of the General Council of Pharmaceutical Colleges of Spain
			#RecomiendoLeer Una voz de peso hablando de #Nutriscore que tanta opinión divergente por la comunidad científica ha generado estas semanas Gracias por compartir @RUrrialde_PhD	“#RecommendRead A strong voice talking about #Nutriscore that has generated so much divergent opinion by the scientific community these weeks Thanks for sharing @RUrrialde_PhD
**T2** Multinational companies against the NS adoption	good Cola also have made Coca should must still Nestlè	9.0%	Todavia faltan otras Coca-cola, Mars, Ferrero, Mondelez, Unilever… Preguntar por qué no añaden #Nutriscore en sus envases. OCU y CECU continuan la lucha para conseguirlo	Other Coca-Cola, Mars, Ferrero, Mondelez, Unilever are still missing… Ask why they don’t add #Nutriscore to their packaging. OCU and CECU continue the fight to achieve it
			Gracias a la presión de las asociaciones de consumidores, se ha conseguido que grandes empresas, como Nestlé, durante años opuestas a #Nutriscore, lo acepten. Preguntar a Coca, Mars, Ferrero, Mondelez, Unilever por qué todavia no lo añaden en sus envases	Thanks to the pressure of consumer associations, large companies such as Nestlé, for years opposed to #Nutriscore, have been able to accept it. Ask Coca, Mars, Ferrero, Mondelez, Unilever why they still don’t add it to their packaging
			Es oficial, Kellogg’s adopta #nutriscore !! Todavia faltan otras multinacionales: Unilever, Mars, Coca, Pepsi, Mondelez… les consumidores esperan que adopten nutriscore rapidamente	It’s official, Kellogg’s adopts #nutriscore !! Other multinationals are still missing: Unilever, Mars, Coke, Pepsi, Mondelez… consumers expect them to quickly adopt nutriscore
**T3** Criticisms towards the NS system	to do bad problem to elaborate know negative put can carry change	6.1%	No alcanzan las letras del alfabeto para lo bajo que puntúan en nutriscore unos alfajores te juro qué tristeza	The letters of the alphabet are not enough for the low nutriscore level of some alfajores I swear how sad
			El nutriscore ha sido creado a medida para que las empresas que se dedican a los procesados no salgan mal paradas. Unos Chocapic tienen una B. Es una vergüenza y solo va a servir para que la gente siga comiendo mal	The nutriscore has been created to measure so that companies that are dedicated to processed foods do not go badly off. Some Chocapic have a B. It’s a shame and it will only serve to keep people eating badly
			“Nutriscore no entra a valorar si un producto es bueno o malo” Lo que verá el usuario medio: A (verde): bueno E (rojo): malo Y lo saben. Nutriscore = basura Idea original medianamente buena. Ejecución PÉSIMA	Nutriscore does not enter to assess whether a product is good or bad” What the average user will see: A (green): good E (red): bad And they know it. Nutriscore = garbage Moderately good original idea. POOR execution
**T4** Research support of the NS	study are major evidence person advertising real thread work interest	9.1%	Casi 60 investigadores de reconocido prestigio firman este interesante artículo. Association between nutritional profiles of foods underlying Nutri-Score front-of-pack labels and mortality: EPIC cohort study in 10 European countries	Almost 60 renowned researchers sign this interesting article. Association between nutritional profiles of foods underlying Nutri-Score front-of-pack labels and mortality: EPIC cohort study in 10 European countries
			Gran estudio europeo (501,000 personas,10 paises y 17 años de seguimiento) publicado en el BMJ que confirma los resultados de otras cohortes y la pertinencia e interés del algoritmo subyacente a #NutriScore por su asociación con la mortalidad y las grandes enfermedades crónicas	Large European study (501,000 people, 10 countries and 17 years of follow-up) published in the BMJ confirming the results of other cohorts and the relevance and interest of the algorithm underlying #NutriScore due to its association with mortality and major chronic diseases
			Estudio. #Nutriscore. Publicado en el BMJ los resultados de un estudio que demuestra que las personas que consumían en promedio más alimentos con menor clasificación por Nutri-Score presentaban un aumento de la mortalidad	Study. #Nutriscore. Published in the BMJ the results of a study that shows that people who consumed on average more foods with lower classification by Nutri-Score had an increase in mortality
**T5** Political slip-ups in the NS adoption	only Garzon same (masculine) thing can good category same (feminine) say compare	11.7%	La diputada del PP Carmen Riolobos llama “vendido” a Garzón por implantar Nutriscore… hasta que descubren que ellos mismos lo exigieron	The deputy of the PP Carmen Riolobos calls Garzón “corrupt” for implementing Nutriscore… until they discover that they themselves demanded it
			La banda criminal @populares llama “vendido” a @garzon por implantar @NutriScore… hasta que descubren que ellos mismos lo exigieron	The criminal gang @populares calls “ corrupt “ @garzon for implementing @NutriScore… until they find out they demanded it themselves
			El lenguaje político actual es bélico: si no estás conmigo, eres un traidor a la patria. Y eso es una absoluta vergüenza. (Al margen de si el etiquetado Nutriscore es bueno o no (aunque se le han visto muchos fallos))	The current political language is warlike: if you are not with me, you are a traitor to the country. And that is an absolute shame. (Regardless of whether Nutriscore labeling is good or not (although many failures have been seen))
**T6** NS calculation: possible chinks in the system	to be so here healthy clear day case two places true	7.9%	Respecto a la cuestión planteada sobre la complementariedad entre #NutriScore y ultra-procesamiento leer el documento “Nutri-Score y ultra procesamiento: dos dimensiones diferentes, complementarias y no contradictorias”	Regarding the question raised about the complementarity between #NutriScore and ultra-processing, read the document “Nutri-Score and ultra-processing: two different dimensions, complementary and not contradictory”
			Una lata de fabada contiene aprox. 80 g de chorizo, morcilla, panceta y manteca de cerdo. Por separado cualquiera de esos ingredientes tienen NutriScore E, pero cuando se cocinan junto a las alubias, el resultado es NutriScore A	A tin of fabada contains approx. 80 g of chorizo, black pudding, bacon and lard. Separately, any of those ingredients have NutriScore E, but when cooked together with the beans, the result is NutriScore A
			Me estoy tomando un batido de frutas riquísimo: zumo de piña, plátano, mango, leche de coco, zumo de limón y de repente me fijo q ya tiene la etiqueta del Nutriscore y le pone mala nota (?). Cómo estamos tragando con estas chorradas americanas?	I am drinking a delicious fruit smoothie: pineapple juice, banana, mango, coconut milk, lemon juice and suddenly I notice that it already has the Nutriscore label and it gives it a bad grade (?). How are we swallowing with this American bullshit?
**T7** NS adoption	label nutrition consumer traffic light quality new frontal information France	21.2%	Unión Europea: La etiqueta NutriScore es efectiva para elegir alimentos saludables	European Union: The NutriScore label is effective for making healthy food choices
			Es necesario mejorar la forma en la que se informa al consumidor sobre la calidad nutricional de lo que consume. El Nutriscore es un método validado e intuitivo. Por eso yo ya firmé para que sea obligatoria su implantación en Europa	It is necessary to improve the way in which consumers are informed about the nutritional quality of what they consume. The Nutriscore is a validated and intuitive method. That is why I already signed so that its implementation in Europe is mandatory
			La Sociedad Francesa de Nutrición (SFN) apoya la Iniciativa Ciudadana Europea PRO-NUTRISCORE lanzada por 7 asociaciones de consumidores para hacer obligatorio el #NutriScore en Europa	The French Society for Nutrition (SFN) supports the European Citizens’ Initiative PRO-NUTRISCORE launched by 7 consumer associations to make the #NutriScore mandatory in Europe
**T8** NS vs. traditional foods	Jamon Iberico to see do it seemed EVOO classification time has to reason	7.1%	El tratamiento del #AOVE como una grasa similar a la colza, es una absoluta insensatez. Revisen por favor estos modelos nutricionales de «corrección alimentaria» cancelación de nuestra cultura gastronómica	Treating the #EVOO as a rapeseed-like fat is absolute nonsense. Please review these nutritional models of “food correction” cancellation of our gastronomic culture
			El jamón ibérico aporta proteínas de alto valor biológico que proporcionan aminoácidos esenciales y lípidos con cierto grado de instauración que favorecen su digestibilidad #Nutriscore #100 × 100 nuestra #YoAceiteyJamon #cerdoiberico #jamon #saludable	Iberian ham provides proteins of high biological value that provide essential amino acids and lipids with a certain degree of establishment that favor its digestibility #Nutriscore #100 × 100 nuestra #YoAceiteyJamon #cerdoiberico #jamon #saludable
			Poco se le ha linchado al NutriScore para lo que se merece. Y cuando un jamón baje su nivel de sal, dejará de ser jamón. Pongamos (consumamos) el jamón como lo que es y dejemos de retorcer la realidad para acomodarla a nuestra conveniencia	NutriScore was less lynched than it deserves. And when a ham lowers its salt level, it will stop being a ham. Let’s put (consume) the ham for what it is and stop distorting reality to adapt it to our liking
**T9** NS calculation: technical aspects	sugar fat ultra-processed cereal salt extra calories amount high neither	9.2%	En cada producto se tienen en cuenta aspectos… Negativos: la cantidad de calorías, azúcares, grasas saturadas y sal, y Positivos: el porcentaje de frutas o verduras empleado para obtener el producto, y su aporte de fibra y proteínas	These Aspects are taken into account in each product...Negatives: the amount of calories, sugars, saturated fats and salt, and Positives: the percentage of fruits or vegetables used to obtain the product, and its contribution of fiber and protein
			Un vaso de Cacaolat Veggie contiene 35,6 g de azúcar, equivalente a 8,9 terrones. NutriScore B	A glass of Cacaolat Veggie contains 35.6 g of sugar, equivalent to 8.9 cubes. NutriScore B
			El nutriscore califica como C al aceite de oliva y eso que lo modificaron. Antes tenía una D. Pero montones de ultraprocesados califican como A	The nutriscore classifies olive oil as C-and they modified it-. It used to have a D. But lots of ultra-processed (products) are classified as A
**T10** NS vs. olive oil (and other traditional products)	olive less industry to eat value oil benefit coke see want	8.3%	La defensa del Ministerio es anular al aceite de oliva. Eso es ayuda? El Ministerio de Consumo defenderá los beneficios nutricionales del aceite de oliva en el Nutri-Score	The Ministry’s defense is to annul (the label on) olive oil. Is that help? The Ministry of Consumption will defend the nutritional benefits of olive oil in the Nutri-Score
			El sector oleícola traslada al ministro de Consumo el problema del NutriScore ‘Considera que minusvalora los beneficios saludables del consumo de aceites de oliva’	The olive sector transfers the NutriScore problem to the Minister of Consumption “It considers that it undervalues the healthy benefits of consuming olive oils”
			✔ En su opinión, dicha clasificación no refleja los #beneficios #nutricionales del aceite de oliva y lo equipara con el de otras grasas como el aceite de colza.	✔ In his opinion, this classification does not reflect the #nutritional #benefits of olive oil and equates it with that of other fats such as rapeseed oil.

## Appendix B

**Table A2:** Topics identified in the scientific literature corpus.

Topic	Most Typical Terms	Prevalence	Exemplary Documents (Title and Reference)	Article Addressing the Topic (Topic Prevalence >25%)
**T1** Impact OF FOP labels on healthy choices	FOP condition * perceive attention without experiment * segment label * online estimation	11.6%	Experimental study of front-of-package nutrition labels’ efficacy on perceived healthfulness of sugar-sweetened beverages among youth in six countries [80] Nutri-Score, multiple traffic light and incomplete nutrition labelling on food packages: Effects on consumers’ accuracy in identifying healthier snack options [87] The use of food swaps to encourage healthier online food choices: a randomized controlled trial [81]	[42,43,44,45,46,47,48,80,81,87,88,89,90,91,92,93,94,95,96,97,98,99,100,101,102,103,104,105]
**T2 **** Advertisements drive unhealthy food choices	advertisment * children companies adolescents commitment television Spain obesity package * value *	9.5%	Food advertising and prevention of childhood obesity in Spain: Analysis of the nutritional value of the products and discursive strategies used in the ads most viewed by children from 2016 to 2018 [70] Soft drinks and sugar-sweetened beverages advertising in Spain: Correlation between nutritional values and advertising discursive strategies [65] Breakfast food advertising and prevention of obesity: Analysis of the nutritional value of the products and discursive strategies used in the breakfast ads from 2015 to 2019 [66]	[18,65,66,67,68,69,70,85,104,106,107,108,109,110,111,112,113,114]
**T3** NS and ultra-processed foods	ultra-processed natur * bars g*reen* cereals UPF (ultra-processed Food) process HMF (hydroxymethylfurfural) degree NOVA (classification)	6.5%	Association between heat-induced chemical markers and ultra-processed foods: A case study on breakfast cereals [115] Naturalness and healthiness in ultra-processed foods: A multidisciplinary perspective and case study [71] Respective contribution of ultra-processing and nutritional quality of foods to the overall diet quality: results from the NutriNet-Santé study [116]	[71,72,83,86,115,116,117,118,119,120,121]
**T4** Different nutrient profiling systems	sale * interpretation scheme warning HSR (Health Star Rating) OFCOM (Office of Communication) PAHO (Pan-American Health Organization) valid store classification	7.5%	Comparison of nutrient profiling models for assessing the nutritional quality of foods: A validation study [62] Food Compass is a nutrient profiling system using expanded characteristics for assessing healthfulness of foods [122] Facilitating consumers choice of healthier foods: A comparison of different front-of-package labelling schemes using Slovenian food supply database [123]	[18,62,63,64,86,98,101,110,122,123,124,125,126,127]
**T5 **** Assessment of nutritional quality of food through the NS	meat price cart cheese shop arm * analogous RIs (Reference Intakes) lower point	8.0%	Plant-Based Alternative Products: Are They Healthy Alternatives? Micro- and Macronutrients and Nutritional Scoring [128] Dietary intake assessment of pre-packed graviera cheese in Greece and nutritional characterization using the Nutri-score front of pack label scheme [129] Assessment of price and nutritional quality of gluten-free products: Versus their analogues with gluten through the algorithm of the Nutri-score front-of-package labeling system [73]	[73,74,75,76,128,129,130,131,132,133,134,135]
**T6** Assessment of NS performance and adherence with dietary guidelines	algorithm grain carbohydrates align guidelines nuts discriminatory component pyramid dietary	10.5%	Evaluation of the ability of Nutri-score to discriminate the nutritional quality of prepacked foods using a sale-weighting approach [57] Alignment of Nutri-Score with Mediterranean Diet Pyramid: A Food Level Analysis [54] Performance of the front-of-pack nutrition label Nutri-score to discriminate the nutritional quality of foods products: A comparative study across 8 European countries [59]	[54,55,56,57,58,59,60,61,126,131,136,137,138,139,140,141,142,143]
**T7** Nutritional evaluation and environmental impact of food products	nutriRECIPE environment burger milk EII (Environmental Impact Index) plant-based beef Eco-Score meal alternative	7.4%	A combined Nutri-Score and ‘Eco-Score’ approach for more nutritious and more environmentally friendly food choices? Evidence from a consumer experiment in Belgium [50] Meat substitution in burgers: nutritional scoring, sensorial testing, and Life Cycle Assessment [144] The nutriRECIPE-Index—development and validation of a nutrient-weighted index for the evaluation of recipes [145]	[7,8,11,49,50,96,103,112,144,145,146,147,148]
**T8** Medical aspects	mortal FSAm-NPS cohort association weight cancer risk dietary FSA-NP hazard	9.7%	Association between nutritional profiles of foods underlying Nutri-Score front-of-pack labels and mortality: EPIC cohort study in 10 European countries [149] Nutritional quality of food as represented by the FSAm-NPS nutrient profiling system underlying the Nutri-Score label and cancer risk in Europe: Results from the EPIC prospective cohort study [82] Food consumption based on the nutrient profile system underlying the Nutri-Score and renal function in older adults [52]	[51,52,53,82,84,116,149,150,151,152,153,154,155,156,157,158]
**T9** NS understanding and policy debates	olive Italian rank oil Italy NS cake behaviour pizza correct	7.6%	Assessing the effectiveness of front of pack labels: Findings from an online randomised-controlled experiment in a representative British sample [159] Legitimacy of Front-of-Pack Nutrition Labels: Controversy Over the Deployment of the Nutri-Score in Italy [10] Is FOP nutrition label Nutri-score well understood by consumers when comparing the nutritional quality of added fats, and does it negatively impact the image of olive oil? [12]	[10,12,34,40,41,79,83,143,159,160,161,162,163,164,165]
**T10** Understanding of different FOP labels	FOPL understand perception multiple reference traffic light trust format star	17.7%	Improving the understanding of key nutritional elements to support healthier and more informed food choices: The effect of front-of-pack label bundles [166] Consumers’ responses to front-of-pack nutrition labelling: Results from a sample from the Netherlands [36] Objective understanding of the Nutri-score front-of-pack label by European consumers and its effect on food choices: an online experimental study [167]	[6,18,33,34,35,36,37,38,78,97,102,105,111,117,142,154,155,160,161,163,164,166,167,168,169,170,171,172,173,174,175,176]

Note: * is used to include plurals, e.g., condition and conditions. ** NS is generally used as a tool to discriminate products according to their nutritional profile; the label has not been tested in some ways.
